# TRPV1 channel in spermatozoa is a molecular target for ROS-mediated sperm dysfunction and differentially expressed in both natural and ART pregnancy failure

**DOI:** 10.3389/fcell.2022.867057

**Published:** 2022-09-23

**Authors:** Nirlipta Swain, Luna Samanta, Chandan Goswami, Sujata Kar, Rakesh Kumar Majhi, Sugandh Kumar, Anshuman Dixit

**Affiliations:** ^1^ Redox Biology & Proteomics Laboratory, Department of Zoology, School of Life Sciences, Ravenshaw University, Cuttack, Odisha, India; ^2^ School of Biological Sciences, National Institute of Science Education and Research, HBNI, Khordha, Odisha, India; ^3^ Centre for Excellence in Environment and Public Health, Ravenshaw University, Cuttack, Odisha, India; ^4^ Kar Clinic and Hospital Pvt. Ltd., Bhubaneswar, India; ^5^ Computational Biology and Bioinformatics Laboratory, Institute of Life Sciences, Bhubaneswar, Odisha, India

**Keywords:** TRPV1, oxidative stress, unilateral varicocele, IVF, ICSI, calcium, acrosomal reaction, embryonic development

## Abstract

Bi-directional crosstalk between Ca^2+^ signaling and ROS modulates physiological processes as a part of a regulatory circuit including sperm function. The role of transient receptor potential vanilloid 1 (TRPV1) in this regard cannot be undermined. This is the first report demonstrating the Ca^2+^-sensitive TRPV1 channel to be under-expressed in spermatozoa of subfertile men, idiopathic infertile men, and normozoospermic infertile males with high ROS (idiopathic infertility and unilateral varicocele). To study the effect of TRPV1 in determining the fertility outcome, we compared the expression profile of TRPV1 in spermatozoa of male partners who achieved pregnancy by natural conception (NC+, *n* = 10), IVF (IVF+, *n* = 23), or ICSI (ICSI +, *n* = 9) and their respective counterparts with failed pregnancy NC (*n* = 7), IVF (*n* = 23), or ICSI (*n* = 10), by both immunocytochemistry and flow-cytometry. Reduced expression of TRPV1 in sperm of IVF ± and ICSI ± men with respect to that NC+ men imply its role in mediating successful fertilization. Unsuccessful pregnancy outcome with an underexpression of TRPV1 in sperm of NC-/IVF-/ICSI-men suggests its role in conception and maintenance of pregnancy. Since ROS is regarded as one of the major contributors to sperm dysfunction, the effect of H_2_O_2_ +/- TRPV1 modulators (RTX/iRTX) on acrosomal reaction and calcium influx was evaluated to confirm TRPV1 as a redox sensor in human sperm. A significant increment in the percentage of acrosome reacted spermatozoa along with augmented Ca^2+^-influx was observed after H_2_O_2_ treatment, both in the presence or absence of TRPV1 agonist resiniferatoxin (RTX). The effect was attenuated by the TRPV1 antagonist iodoresiniferatoxin (iRTX), indicating the involvement of TRPV1 in mediating H_2_O_2_ response. Enhancement of motility and triggering of acrosomal reaction post TRPV1 activation suggested that disruption of these signaling cascades *in vivo*, possibly due to down-regulation of TRPV1 in these subfertile males. Bioinformatic analysis of the crosstalk between TRPV1 with fertility candidate proteins (reported to influence IVF outcome) revealed cell death and survival, cellular compromise, and embryonic development to be the primary networks affected by anomalous TRPV1 expression. We therefore postulate that TRPV1 can act as a redox sensor, and its expression in spermatozoa may serve as a fertility marker.

## Introduction

The role of reactive oxygen species (ROS) (Aitken and Drevet, 2020) and calcium in sperm function is well established (Jimenez-Gonzalez et al., 2006) where physiological level drives important sperm functions such as capacitation, acrosome reaction, fertilization, and augmented level results in DNA fragmentation, change in membrane fluidity, apoptosis and failed fertilization. With the silencing of transcriptional and translational machinery in spermatozoa, maintenance of ion homeostasis particularly calcium and redox balance in a spatiotemporal manner is essential to mediate successful fertilization (Suarez, 2008; Beltrán et al., 2016; Puga Molina et al., 2018). Although data on spermatozoa in general and with respect to humans are unavailable, bi-directional crosstalk between Ca^2+^-signaling and ROS modulates physiological processes as a part of a regulatory circuit is reported (Görlach et al., 2015). High levels of ROS increase Ca^2+^-influx into cells by modulating the activity of versatile Ca^2+^ channels, pumps, and exchangers. Subsequently, Ca^2+^-overload in cells activates ROS-generating enzymes augmenting free radical production (Görlach et al., 2015). At physiological levels, such reciprocal interaction generally functions as a compensatory mechanism to maintain the redox balance and Ca^2+^-homeostasis of the cell. However, at pathological levels of either Ca^2+^ and/or ROS, the entire feedback cycle would essentially get disturbed thereby potentially damaging the cell, and ultimately triggering apoptosis (Feno et al., 2019). Thus, it could be surmised that ROS regulates the function of the intracellular calcium channels, which in turn control ROS levels. With regard to spermatozoa, studies have only speculated indirect activation of calcium channels in sperm by ROS *via* activation of kinases and subsequent phosphorylation of the channel. However, several ion channels can be directly activated by ROS to modulate the pathophysiological conditions. One such versatile yet less explored redox-sensitive ion channel in human sperm is the transient receptor potential vanilloid 1 (TRPV1) channel (Francavilla et al., 2009; Lewis et al., 2012; De Toni et al., 2016). TRPV1 is a multimodal calcium conducting ion channel belonging to the mammalian TRP channel family, whose activity in regulating sperm capacitation and acrosomal reaction has been demonstrated in different species (Maccarrone et al., 2005; Francavilla et al., 2009; Bernabò et al., 2010; Cobellis et al., 2010; Catanzaro et al., 2011; Gervasi et al., 2011; Lewis et al., 2012; Osycka-Salut et al., 2012; De Toni et al., 2016; Kumar et al., 2019). However, most of the studies of TRP in human spermatozoa have been undertaken with fertile and/or normozoospermic individuals, with few data as yet available on the activity and expression of TRP in spermatozoa of males with known or idiopathic infertility. Recently, Zhu et al. (2018) reported reduced expression of TRPC5 in patients with varicocele-associated asthenozoospermia.

Albeit, the physiological relevance of ROS being demonstrated as a regulatory factor for sperm capacitation and acrosome reaction (Oehninger et al., 1995; Hsu et al., 1999; Rivlin et al., 2004), the underlying factors involved in the ROS-mediated events need further analysis. The bi-directional modulation of TRPV1 and ROS in mediating cell injury and/or death has been reported in other systems (Choi et al., 2014; Ogawa et al., 2016). Due to the paucity of information on the functioning of TRP channels under oxidative stress conditions in spermatozoa, it is explored in the present study by combining *in vivo*, *in vitro*, and *in silico* approaches. The experiments were designed in three phases with regard to the expression of functional TRPV1 channels in human spermatozoa. In the first phase, TRPV1 expression was correlated with pregnancy outcome both under natural conditions and assisted reproduction, while in the second phase, it was studied in conditions where spermatozoa are subjected to oxidative predominances such as semen with high ROS, varicocele, or spermatozoa with a morphological anomaly or maturation arrest. In addition, varicocele is a unique condition where abnormal distension of the pampiniform venous plexus that circumvents the venous drainage from the testes result in hypoxic and hyperthermic conditions, leading to oxidative stress (Samanta et al., 2018). In the third phase, a novel approach was undertaken to study the effect of hydrogen peroxide (H_2_O_2_) in presence of TRPV1 modulators in the context of acrosomal reaction and Ca^2+^-influx in sperm. The effect of channel modulators on sperm functions in oxidative stress conditions further implied the relevance of TRPV1 in determining sperm quality and fertility potential. Finally, *in silico* analysis of the disease and function networks affected by TRPV1 in conjunction with curated differentially expressed proteins (DEPs) known to be associated with pregnancy outcome post assisted reproductive technology (ART) was carried out to give a better understanding of the role of TRPV1 in spermatozoa.

## Materials and methods

### Sample collection

**FIGURE 1:**
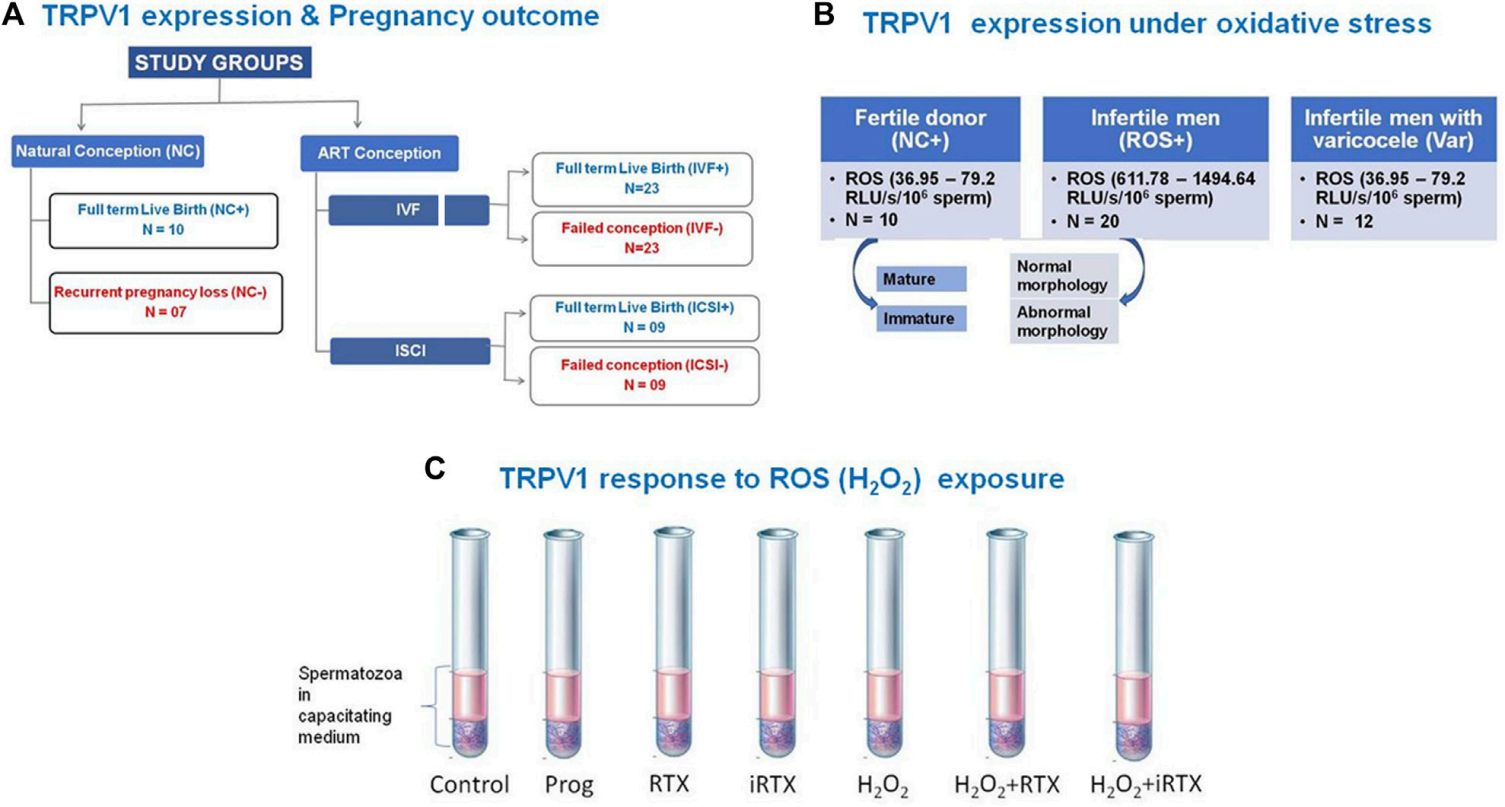
Schematic representation of experimental design including patient selection. **(A)** Expression of TRPV1 in spermatozoa as a function of pregnancy outcome by natural conception and assisted reproductive technology (ART); **(B)** expression of TRPV1 in spermatozoa as a function of altered redox status; **(C)** validation of regulation of TRPV1 function in human spermatozoa by ROS *in vitro*. IVF, *in vitro* fertilization; ICSI, intracytoplasmic sperm injection; ROS, reactive oxygen species; Prog, progesterone, RTX, resinferatoxin; iRTX, 5iodoresinferatoxin; RLU, relative light units.

Semen samples were collected (with informed consent) from idiopathic infertile males, infertile men presented with varicocele males, male partners of patients enrolled for ART treatment, and male partners of recurrent pregnancy loss (RPL) patients (who lost ≥2 clinical pregnancies within the first trimester (Practice Committee of the American Society for Reproductive, 2012). In addition, fertile males (*n* = 10) whose partners achieved pregnancy by natural means within 1 year prior to enrolment in the study were also included (Figure 1). The study was conducted after due approval by the Institutional Ethics Committee of Kar Clinic and Hospital Pvt. Ltd., Bhubaneswar, India.

### Subject selection (Inclusion and exclusion criteria)

This is a prospective case-controlled study that recruited infertile males reporting at the IVF Centre, Kar Clinic and Hospital Pvt. Ltd., Bhubaneswar, Odisha, India for fertility treatment. The following groups of patients were recruited for *in vivo* experiments.

#### Phase-I experiment: Expression of TRPV1 in spermatozoa as a function of pregnancy outcome by natural conception and ART

1) NC+ group: Conception by natural means (men who fathered a child within 1 year, i.e., fertile donors)
2) NC- group: Partners of recurrent pregnancy loss (RPL) patients, that is, who conceived naturally but could not have a live birth
3) IVF+ group: Patients who have live birth after *in vitro* fertilization (IVF; *n* = 23)
4) IVF- group: Patients who failed to conceive even after IVF (*n* = 23)
5) ICSI+ group: Patients who have live birth after intracytoplasmic sperm injection (ICSI; *n* = 9)
6) ICSI- group: Patients who failed to conceive even after ICSI (*n* = 9)

#### Phase-II experiment: Expression of TRPV1 in spermatozoa as a function of altered redox status

##### Experiment—1 comparison of fertile donors having low ROS levels with infertile patients having high ROS levels

1) Fertile group: As mentioned above for NC+
2) ROS+ group: Idiopathic infertile men with high seminal ROS as per the published literature (Agarwal et al., 2015). ROS was measured in the semen by the luminol-dependant chemiluminescence assay with the Berthold luminometer and expressed as relative light units (RLU) per second per million spermatozoa. Greater than 102.2 RLU/s/10^6^ sperm is designated as ROS+ (*n* = 20). Detailed methodology is provided in Supplementary File SI.
3) Var group: Infertile men presented with varicocele based on scrotal clinical examination and further confirmation by color Doppler ultrasonography (*n* = 12).

##### Experiment–2: Expression of TRPV1 in spermatozoa as a function of sperm maturation

1) Immature fraction: Immature spermatozoa isolated from fertile donors as described above (*n* = 10)
2) Mature fraction: Mature spermatozoa isolated from fertile donors as described above (*n* = 10)

##### Experiment–3: Expression of TRPV1 in spermatozoa as a function of sperm morphology

In this experiment, spermatozoa from all the infertile groups were analyzed (*n* = 3 per group randomly selected) and compared with the mature fraction from the fertile donor group.

Clinical pregnancy was diagnosed by ultrasonographic evidence of intrauterine fetal heartbeat at 7 weeks. All the donors selected were aged between 25 and 40 years and normozoospermic according to WHO 2010 criteria (WHO, 2010). Leukocytospermic and samples positive for infections such as toxoplasma, HIV, and herpes virus were excluded. The female partners of the couples were <38 years with normal body mass index (BMI) and lacked any known female factor abnormalities such as chromosomal abnormalities, endocrine disease, endometriosis, or polycystic ovary syndrome (PCOS).

#### Phase-III experiment: Validation of regulation of TRPV1 function by ROS *in vitro*

The actively motile sperm samples from the semen ejaculate of fertile males were divided into the following seven groups.

1) Negative control: Spermatozoa incubated with only capacitation buffer (Quinn’s medium with human serum albumin).
2) Positive control: Spermatozoa in capacitation buffer supplemented with progesterone (10 µM, Sigma Chemical Co., St Louis) (Kumar et al., 2016)
3) Experimental positive control: Spermatozoa in capacitation buffer supplemented with TRPV1 agonist resiniferatoxin (RTX, 1 µM, #R8756, Sigma Chemical Co., St Louis) (Francavilla et al., 2009);
4) Experimental negative control: Spermatozoa in capacitation buffer supplemented with TRPV1 antagonist 5′iodoresiniferatoxin (iRTX, 1 µM, #I9281, Sigma Chemical Co., St Louis) (Barbonetti et al., 2010)
5) Experimental oxidant induction group: Spermatozoa in capacitation buffer supplemented with H_2_O_2_ (10 µM, Sigma Chemical Co., St Louis) (Oehninger et al., 1995).
6) Experimental oxidant induction group with TRPV1 agonist: Spermatozoa in capacitation buffer supplemented with H_2_O_2_ and RTX (concentration as mentioned above).
7) Experimental oxidant induction group with TRPV1 antagonist: Spermatozoa in capacitation buffer supplemented with H_2_O_2_ and iRTX (concentration as mentioned above).

Samples from all groups were incubated for 30 min at 37°C in a water bath, and the samples were taken immediately for evaluating acrosomal reaction and Ca^2+^-imaging. The experiment was repeated 5 times and each reading was taken in duplicate.

### Semen sample processing and analysis

Semen samples were collected by masturbation after 2–5 days of sexual abstinence. Samples were allowed to liquefy completely for 20 min at 37°C before further processing. Basic semen analysis was undertaken according to the WHO guidelines (WHO, 2010), and samples with round cells concentration >1 × 10^6^/ml were discarded. Manual semen analysis was performed using a Makler chamber (Makler, 1980) to determine sperm concentration and motility. Viability was assessed by eosin–nigrosin staining. The sample was mixed with eosin and nigrosin in 1:2:3 ratios, and 10–20 µl was pipetted on two slides and smeared. The live cells and dead cells were stained as white and pink, respectively, and thus the percentage of live cells was calculated.

The samples were processed by the density gradient sperm processing method, where 90% and 45% discontinuous density gradients (AllGrad^®^) were used. 1 ml of the 90% AllGrad^®^ gradient solution was placed in the bottom of a conical tube followed by layering of 1 ml of 45% gradient and 1 ml of the semen sample, respectively. Subsequently, the tube was centrifuged for 20 min at 300 x g. The mature sperm cells accumulated as a pellet at the bottom of the tube while the immature sperm cells were retrieved from the interface between the 45% and 90% gradient. The fraction containing the immature cells was carefully removed to prevent any contamination, washed with sperm washing media (SWM, Quinn’s™, CooperSurgicals^®^) for 10 min at 300 x g, and resuspended in SWM for further processing. The pellet containing the mature sperm cells was further washed once in SWM for 5 min and finally, 1 ml of SWM was gently layered over the undisturbed pellet. Samples were incubated for 1 h at 37°C in 5% CO_2_ in air in a slanting position. The supernatant containing the highly concentrated motile sperm was carefully aspirated and sperm concentration was adjusted to 50 million motile sperm/ml for experimental assays. Both the mature and immature sperm cells were taken for TRP expression studies. The mature and motile spermatozoa were taken for insemination of oocytes either by IVF or ICSI.

### Immunocytochemistry

Immunocytochemical analysis of sperm cells was performed as described previously (Majhi et al., 2013). Spermatozoa isolated from seminal plasma post liquefaction as mentioned above were fixed with paraformaldehyde (final concentration 2%). After fixing the cells with PFA, the cells were permeabilized with 0.1% Triton X-100 in PBS (5 min). Subsequently, the cells were blocked with 5% bovine serum albumin for 2 h. After removing the blocking buffer cells were incubated overnight with TRPV1 primary antibody (Alomone Labs, #ACC-030) at 4°C with constant shaking. The TRPV1 antibody was used with a dilution of 1:200 in phosphate buffer saline supplemented with 0.1% Tween-20 (PBST) containing 1% BSA. To validate the non-specific binding of the primary antibody, the immunizing peptide blocking experiment was performed, where the antibody was pre-incubated with the control peptide antigen for 15 min prior to exposing the cells. After extensive washings, the detection was carried out using appropriate AlexaFluor-488 labeled anti-rabbit (Invitrogen) antibody at 1:1,000 dilutions. Cells were counterstained with 4′,6-diamidino-2-phenylindole (DAPI) for nuclear staining. All images were taken on a confocal laser-scanning microscope (LSM-780, Zeiss) with a 63X-objective and analyzed with the Zeiss LSM image examiner software. Regions of interest (ROIs) were defined using a freehand selection tool for the sperm shape on a differential interference contrast (DIC) image. Noise normalization was carried out *via* the Rolling ball background correction command of Fiji software (free software developed by NIH) using an image-specific radius to define the sliding paraboloid (default is fixed at 50 pixels). The background corrected image was selected, and corrected for thresholding by adjusting the Brightness/Contrast until the entire background is blue (using HiLo Lookup Tables). Thresholding is done to distinguish the background from the foreground. For intensity measurement, the confocal images were converted to a rainbow palate in the Zeiss LSM image browser software where red is the most intense and blue is the least intense. After background correction, the intensity (integrated density) of single sperm cells (acquired at zoom 4) was quantified (Supplementary File SI). Corrected total cell fluorescence (CTCF) was calculated using the following formula: CTCF = integrated density − (area of selected cell × mean fluorescence of background readings). For normalization, a region with no fluorescence next to a sperm cell with the same ROI was taken as background. At least 100 spermatozoa were quantified from each individual and fluorescence intensity measurements of all spermatozoa per group were taken for comparison.

### Evaluation of the acrosomal reaction

The acrosomal status of the spermatozoa was evaluated after staining with fluorescein isothiocyanate-conjugated *Pisum sativum* agglutinin (FITC-PSA), according to Agarwal et al. (2016) with little modifications. FITC-PSA is a lectin that recognizes glycoconjugates in the acrosomal matrix. The staining of only the equatorial segment denotes spermatozoa with absence or perforated acrosome, while staining of the entire acrosome denotes acrosome-intact spermatozoa.

Briefly, an aliquot of 10 µl of sperm suspensions post-treatment was smeared, allowed to air-dry for 15 min, and dehydrated with 95% ice-cold ethanol for 30 min at room temperature. The slides were rinsed twice in PBS for 5 min and subsequently air dried. The slides were then incubated in dark for 30 min at room temperature with FITC-PSA, (#L0770, Sigma chemicals) at a final concentration of 100 µg/ml in PBS. FITC-PSA labeled slides were rinsed with distilled water and then fixed with 2% paraformaldehyde (PFA) for 15 min in dark. After washing thoroughly with distilled water, slides were mounted and images were captured using a confocal laser-scanning microscope (LSM-780, Zeiss) with a 63X-objective under a scan zoom 1. A minimum of 100 spermatozoa per sample were imaged and the percentage of acrosome-reacted sperm was quantified. Spermatozoa with doubtful acrosomal status were discarded from the calculation.

### Ca^2+^-imaging and intensity calculation of sperm cells

Sperm fractions were incubated with Fluo4-AM (10 μM) for 30 min in dark at 37°C in a water bath for loading of the fluorophore dye. Then the labeled spermatozoa were divided into several treatment groups (Phage—III experiments mentioned above). Subsequently, an aliquot of ∼20 μl of the labeled spermatozoa was placed onto the live cell chamber coverslip, and chambers were then mounted on a confocal laser-scanning microscope. Calcium imaging was undertaken with 63X-objective using 488 nm laser, and fluorescence changes were monitored in a time-lapse imaging mode for 400 s at 0.6 scan zoom. The cell suspension was added to the live cell chamber for Ca^2+^-imaging and time series images were acquired in every 5-s interval. Intensity quantification of Ca^2+^-influx was undertaken using Fiji software (Supplementary File SI). The image is converted to 8-bit grayscale, uneven background was removed using the Rolling ball plugin. The background corrected image was selected and automatic thresholding correction was carried out using the menu command: Image/Adjust/Threshold and the threshold was adjusted in B/W mode to remove background noise. For automatically separating or cutting apart particles that touch each other, watershed segmentation was undertaken using the menu command: Process/Binary/Watershed. The entire image was selected by the rectangle selection tool and added as ROI. The intensity of all the frames was calculated with the menu command: Tool/ROI manager/More/multimeasure.

Images were represented in artificial rainbow color with a pseudo scale (red and blue indicating the highest and lowest levels of Ca^2+^, respectively).

### Flow cytometry

The sperm cells were tagged with the primary antibody as described in the aforementioned protocol for immunocytochemistry. However, during post-secondary antibody incubation, the cells were washed and re-suspended in PBS supplemented with 2% BSA and 0.1% sodium azide. Unstained cells were used as a negative control. All fluorescence signals of labelled spermatozoa were analyzed by a FACS Calibur flow cytometer (BD Biosciences). Fluorescence intensities of 10,000 sperms were examined for each assay. The sperm population was gated to exclude debris and aggregates based on low forward scatter and side scatter measurements. Alexa Fluor-488 signal was detected the in FL1 channel. The percentage of AF-488-positive cells and the mean fluorescence intensity (MFI) of the Alexa Fluor-488 signal were calculated and analyzed by using Cell Quest Pro software (BD Biosciences).

### Curation of candidate proteins regulating fertility outcome post ART along with in silico analysis with TRPV1

A Pubmed/Medline^®^ database search for original research articles (last computerized search on 4 March 2021), was undertaken using keywords “human,” “spermatozoa,” “IVF,” “ICSI,” “ART,” “*In vitro* fertilization,” “Intracytoplasmic sperm injection,” “Assisted Reproductive Technologies,” and “Proteomics.” Articles involving human studies were only incorporated. Data that were solely published in conference or meeting proceedings, websites, or books were excluded. Case reports and reviews were also collected to screen their reference lists for additional relevant articles. For articles published in languages other than English, the abstracts in English language only were accessed. Full texts were retrieved through either journal access from the library or a request to the author. The studies which reported the differentially expressed proteins (DEPs) in IVF or ICSI spermatozoa samples as determined by proteomic strategies were only considered.

Protein–protein interactions such as physical and genetic interactions, co-expression, and colocalization of the selected proteins with TRP proteins were probed via GENEMANIA plugin V_3.5.0 (Warde-Farley et al., 2010) in Cytoscape software V_3.6.1. The known reported interactions were only considered for analysis. In order to further investigate the protein interaction and association of the TRP channels in key biological pathways, these DEPs along with TRPV1 were subjected to Ingenuity Pathway Analysis (IPA) (Ingenuity Systems, http://www.ingenuity.com, content version 47547484, release date: 08-02-2019) was used.

### Statistical analysis

Data are represented as mean ± standard error of the mean. The Shapiro-Wilk test was used to assess data normality and Levene’s test for homogeneity of variance. For comparison of channel immunocytochemical expression in different categories of pregnant and non-pregnant groups, the Kruskal–Wallis test was used, while for FACS data, one-way ANOVA followed by Tukey’s HSD post-hoc test was used. Likewise, for channel expression comparison between mature and immature sperm fractions, the Mann–Whitney test was used. For significance testing of the effect of channel modulators on motility, acrosomal reaction, and calcium influx, one way ANOVA was used followed by Tukey’s HSD post-hoc test. All analyses were done using IBM-SPSS software V_21, and *p* < 0.05 was considered significant. Trellis plot and swarm plots were created using Origin Pro 2017 and the R Project for Statistical Computing software, respectively.

## Results

### Expression of TRPV1 in spermatozoa as a function of pregnancy outcome by natural conception and ART

**FIGURE 2:**
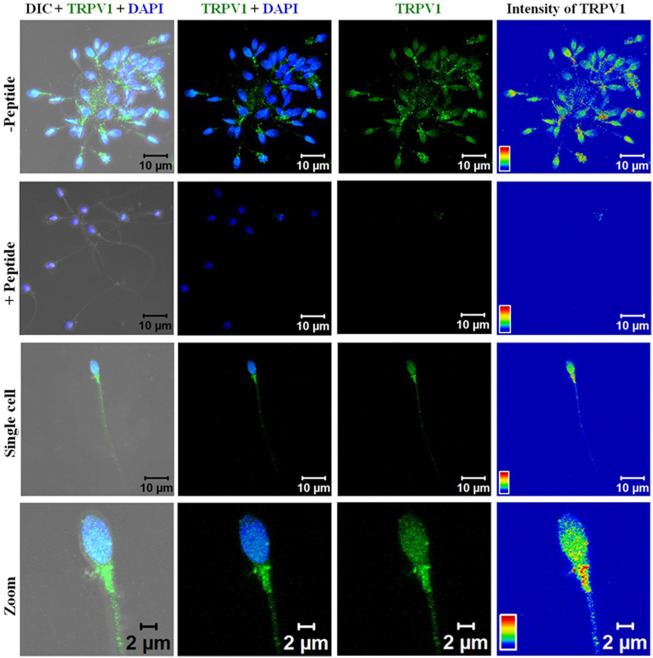
Representative confocal photomicrograph (63X) showing immunocytochemical localization and expression profile of TRPV1 channel (green) in human sperm. First row (peptide-) sperm cluster in absence of immunizing peptide showing the positive staining of the channel (green) and counter stained with DAPI; second row (peptide+): sperm cluster in presence of immunizing peptide (blocking the primary antibody) showing spermatozoa unstained for TRPV1 validating the antibody; third row: single sperm; and fourth row: zoomed view of single sperm showing the distribution pattern of the channel); right column showing the intensity calculation. DIC: differential interference contrast; DAPI: 4′,6-diamidino-2-phenylindole (blue nuclear counter stain). TRPV1 primary antibody (Alomone Labs, #ACC-030) was used at 1:200 dilution. Confocal images were obtained using argon-ion laser (excitation: k 488 nm) and the k 405 nm diode laser for DAPI. Calibration bars are given within each image.

The expression of the TRPV1 channel was confirmed using the control peptide against the antibody used for the assay where the sperm cells from the fertile donor had TRPV1 expression throughout their body covering the head, midpiece, and the tail (Figure 2). The specificity of the antibody was corroborated by ICC and FACS data using the control peptide (Supplementary Figure S1).

**FIGURE 3:**
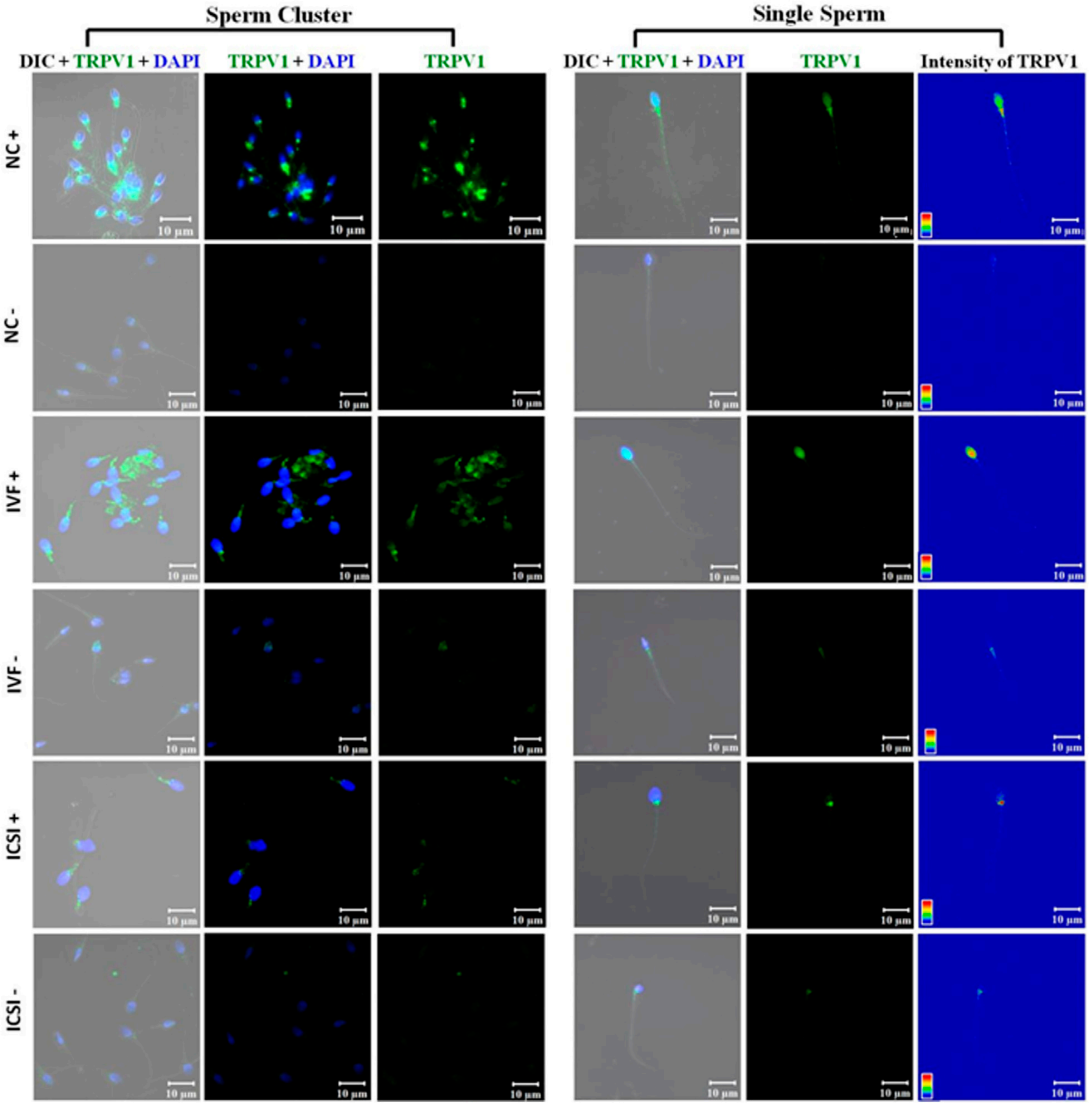
Representative confocal photomicrograph (63X) of TRPV1 expression (green) counter stained for nucleus with DAPI (blue) in spermatozoa of male partners of couples with different pregnancy outcomes. Couples with successful pregnancy by natural conception (NC+), by IVF (IVF+), or by ICSI (ICSI +) were compared to recurrent pregnancy loss (RPL) couples by natural conception (NC-) or with failed pregnancy after IVF (IVF-) or ICSI (ICSI-). Left panel shows sperm clusters and right panel shows single sperm from the corresponding cluster. Calibration bars are given within each image.

**FIGURE 4:**
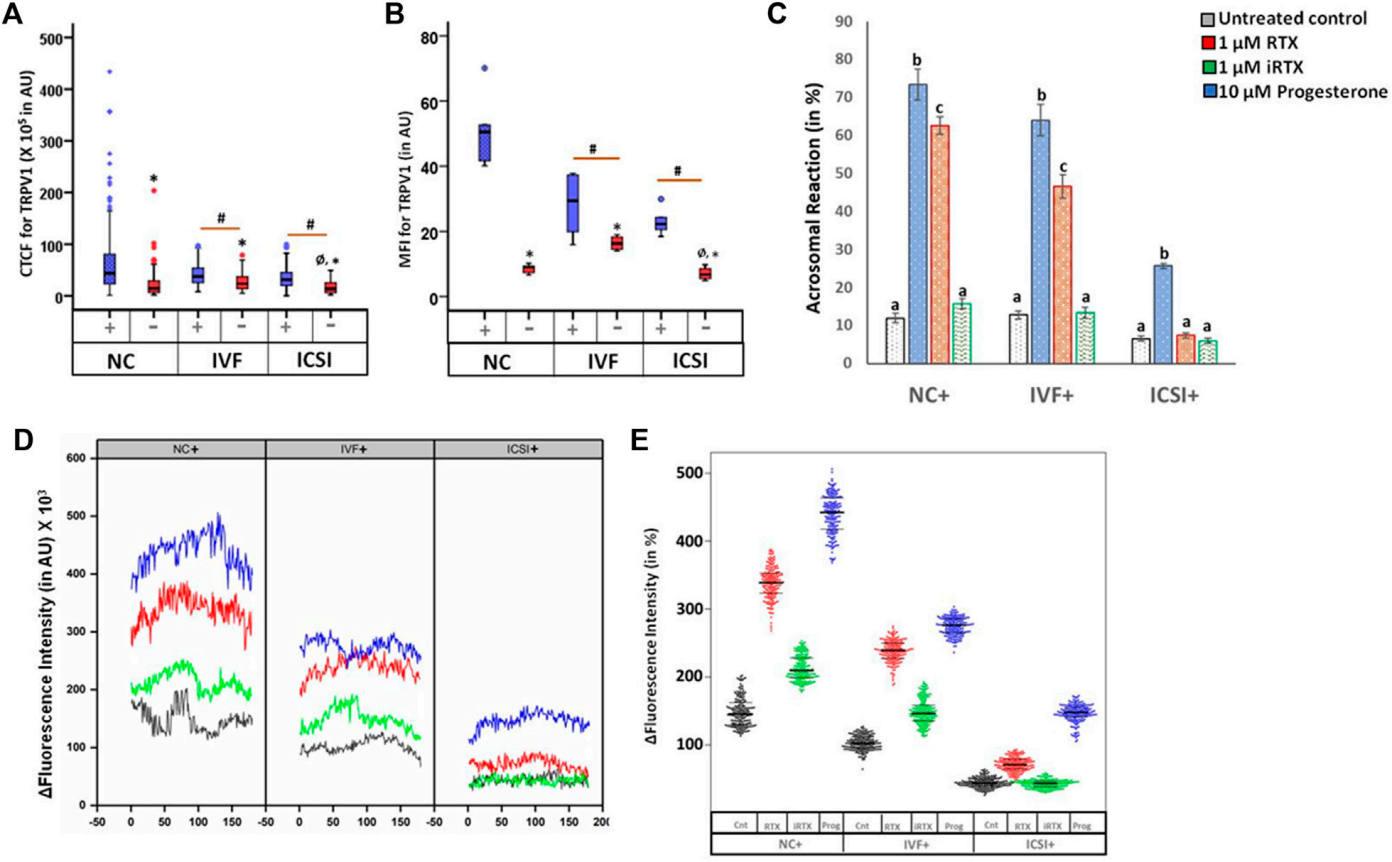
Expression profile of TRPV1 in spermatozoa of male partners of couples with different pregnancy outcomes. Couples with successful pregnancy by natural conception (NC+), by IVF (IVF+), or by ICSI (ICSI +) were compared to RPL couples (NC-), or with failed pregnancy after IVF (IVF-) or ICSI (ICSI-). **(A)** Box-whisker plot of corrected total cell fluorescence (CTCF) intensity of TRPV1 in spermatozoa of different groups in arbitrary units (AU); **(B)** box-whisker plot of mean fluorescence intensity (MFI) of FACs analysis showing the intensity of TRPV1 in spermatozoa of different groups in arbitrary units (AU). MFI data are expressed as geometric mean in logarithmic scale of corresponding histogram. *p < 0.01 with respect to spermatozoa of men with an unsuccessful pregnancy outcome (NC-/IVF-/ICSI-) with respect to their successful counterparts (NC+/IVF+/ICSI+); #*p* < 0.01 with respect to spermatozoa of NC + men; Ø *p* < 0.01 with respect to spermatozoa of IVF + men. **(C)** Percentage of spermatozoa that has undergone acrosome reaction in presence of positive inducer progesterone, resinferatoxin (RTX; agonist of TRPV1 channel), and 5′iodiresinferatoxin (iRTX, antagonist of TRPV1 channel). Data having superscripts of different letters are significantly different from each other at *p* < 0.05. **(D)** Quantification of Fluo-4-AM intensity (in AU) over 500 frames, with the initial value normalized at 100%. Increased fluorescence intensity is observed in most cells, which declines gradually over time. **(E)** Trellis plot demonstrating that calcium fluctuations due to channel activation and inhibition was same for all the fertile males (NC+, IVF+, ICSI +).

The spermatozoa in fertile (NC+) and sub-fertile males who achieved pregnancy after ART (IVF+/ICSI+ group), demonstrated TRPV1 expression all over the body (i.e., head, mid-piece, and tail) while in a few cells it was localized mostly in the head region. An in-depth intensity analysis revealed that the NC+ group has an overall higher intensity in all the three regions as compared to IVF+ and ICSI+. However, in NC+ spermatozoa intensity level was of the order of mid-piece > head > tail, while in IVF+ highest intensity was observed in the head region, and in ICSI+, it was in the mid-piece. On the other hand, a reverse pattern was observed in patients with pregnancy failure by both natural (NC-) and ART (IVF-) conception. In the case of ICSI-, a completely different pattern was noticed where the localization is predominantly in the neck (connecting piece) (Figure 3). When the intensity of expression of the channel was compared, all fertile groups (NC+, IVF+, and ICSI+) have significantly higher levels of expression than the pregnancy failure cases (NC-, IVF- and ICSI-) (Figure 4A) which was further corroborated by FACS data (Figure 4B). Furthermore, the NC + group has the highest level of TRPV1 expression as revealed by both immunocytochemistry and FACS (Figures 4A, B).

The expression of TRPV1 in these patients was further validated by studying the acrosome reaction and Ca^2+^ influx in presence of agonist (RTX) and antagonist (iRTX) and compared with progesterone-induced reactions. As expected, progesterone induced maximum acrosome reaction in the fertile (NC+) and sub-fertile (IVF+ and ICSI+) spermatozoa. RTX was able to induce significant acrosome reaction in NC+ and IVF+ groups though at a reduced level than progesterone, but failed to do so in ICSI+ (Figure 4C). A similar pattern was observed for Ca^2+^ influx in all these groups (Figures 4D, E).

### Expression of TRPV1 in spermatozoa as a function of altered redox status

All the infertile groups included have a higher than normal range of ROS (>102.2 RLU/s/10^6^ spermatozoa) (Figure 1B).

**FIGURE 5:**
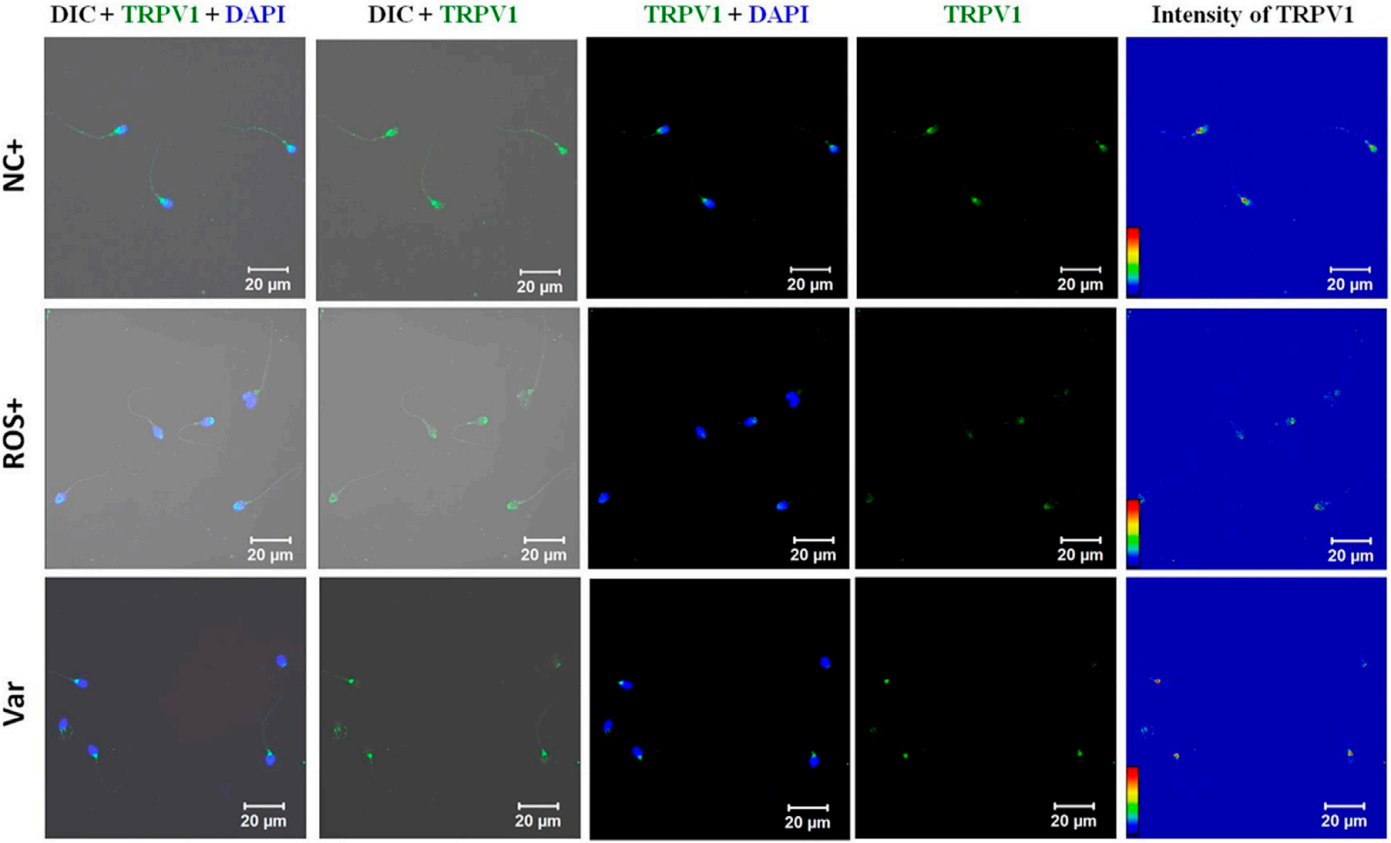
Representative confocal photomicrograph (63X) of TRPV1 localization and expression profile in spermatozoa of fertile donors (NC+), idiopathic infertile men with high ROS (>102.2 RLU/s/10^6^ sperm) (ROS+), and infertile men presented with varicocele (Var+).

In infertile males with known oxidative stress conditions (ROS+, Var), a prominent localization was observed in the head with comparatively reduced expression in the tail region (Figure 5). However, in ROS + spermatozoa, the intensity was maximum in the acrosomal region, while that for varicocele (Var) is a post-acrosomal region (Figure 5). Both ICC and flow cytometry showed significantly decreased expression of TRPV1 in ROS+ and Var groups; nevertheless, no difference was found between the experimental groups (Figure 7A).

**FIGURE 6:**
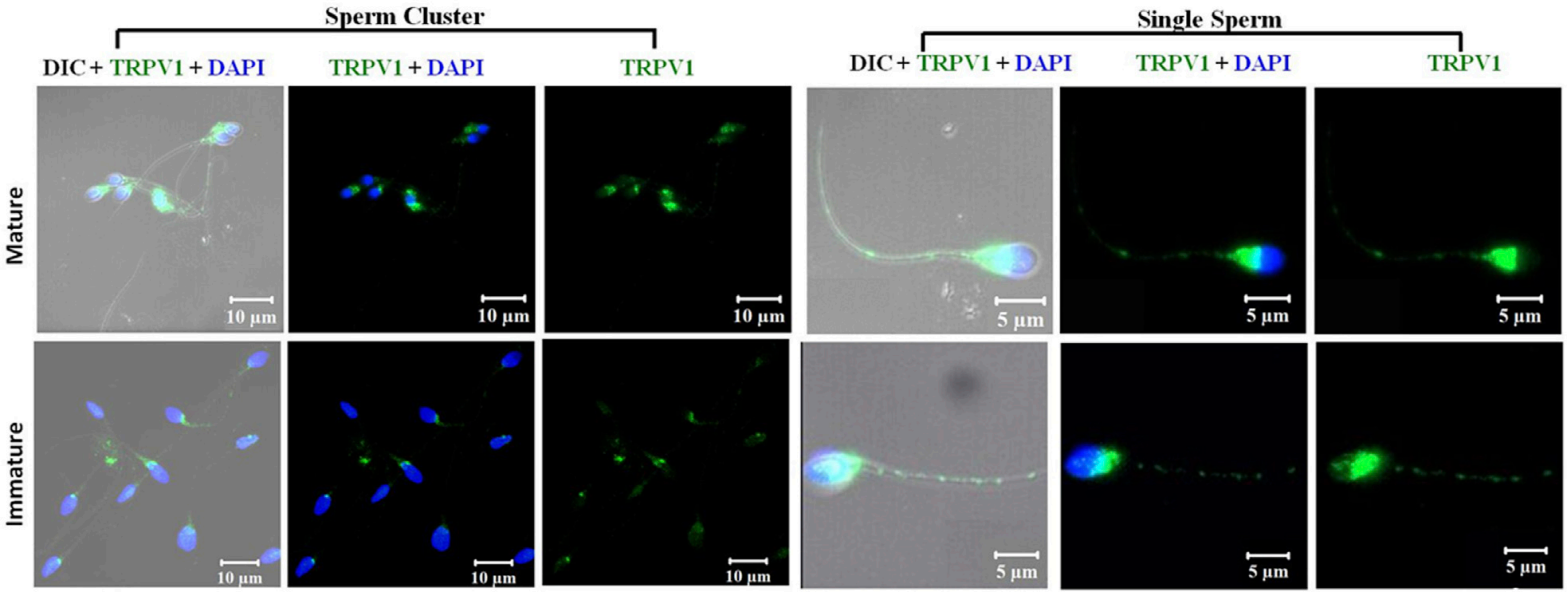
Confocal photomicrograph (63X) showing immunocytochemical localization and expression profile of TRPV1 channel in sperm cluster and single spermatozoa of mature and immature fractions of spermatozoa in fertile donors.

**FIGURE 7:**
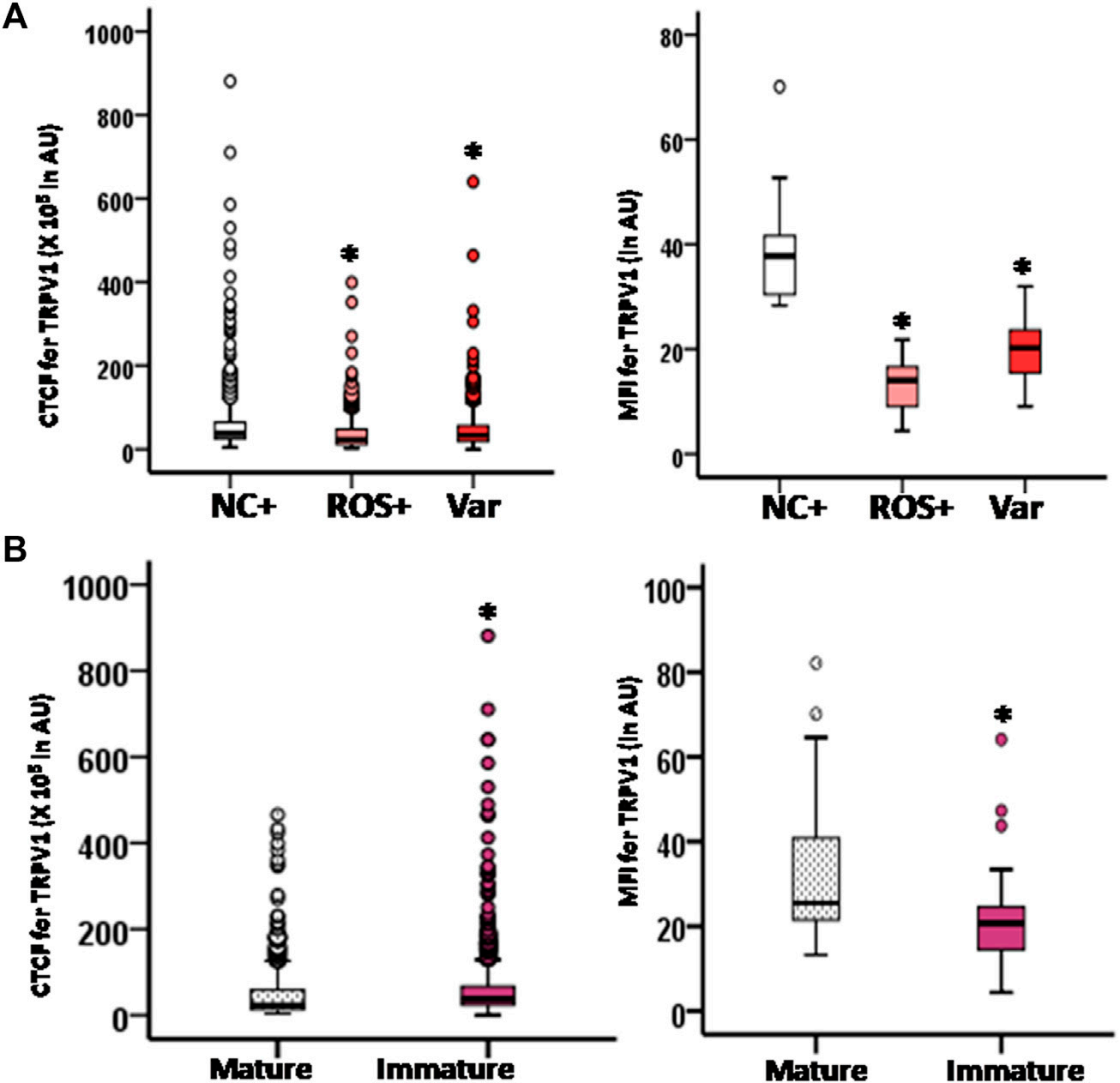
Box-whisker plot of corrected total cell fluorescence (CTCF) intensity of TRPV1 in spermatozoa of different groups in arbitrary units (AU), and box-whisker plot of mean fluorescence intensity (MFI) of FACs analysis showing the intensity of TRPV1 in spermatozoa of different groups in arbitrary units (AU). MFI data are expressed as geometric mean in logarithmic scale of corresponding histogram. **(A)** Expression profile in spermatozoa of fertile donors (NC+), idiopathic infertile men with high ROS (>102.2 RLU/s/10^6^ sperm) (ROS+) and infertile men presented with varicocele (Var+); **(B)** expression profile of TRPV1 channel in spermatozoa of mature and immature fractions of spermatozoa in fertile donors. *p < 0.05.

A comparison between mature and immature fractions demonstrated no change in the TRPV1 localization pattern (Figure 6). The percentage of spermatozoa positive for these TRP channels was significantly decreased in the immature fraction (35.75 ± 6.02% for TRPV1) as compared to the mature fraction (77.10 ± 12.52% for TRPV1) when measured by FACS. However, the intensity of expression was higher in immature sperms when measured by ICC, but declined when measured by FACS (Figure 7B).

**FIGURE 8:**
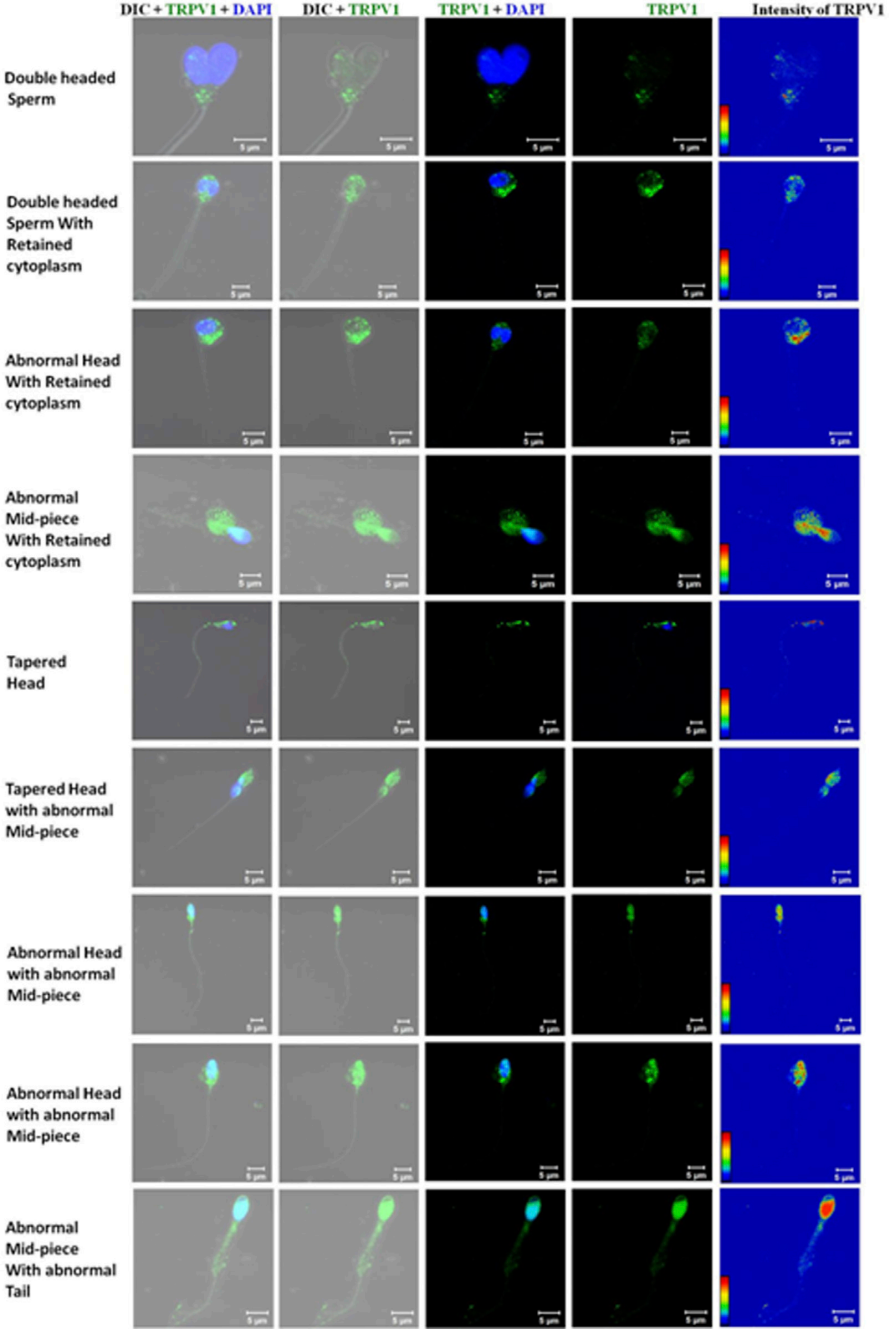
Representative confocal images (63X) showing the expression pattern of TRPV1 in different types of abnormal spermatozoa (morphological abnormality in one of more regions like head, mid-piece, or tail). Calibration bars are given within each image.

Morphologically abnormal spermatozoa had an upregulated localization of the TRPV1 channel where a conspicuous amount of cytoplasm is available as observed under a microscope (swollen regions seen in both DIC and confocal images). Prominent expression of TRPV1 was found in the head and neck region of different types of sperm abnormalities such as spermatozoa with double head, tapered head, and abnormal mid-piece (Figure 8).

### Validation of regulation of TRPV1 function by ROS *in vitro* (TRPV1 as a redox sensor)

**FIGURE 9:**
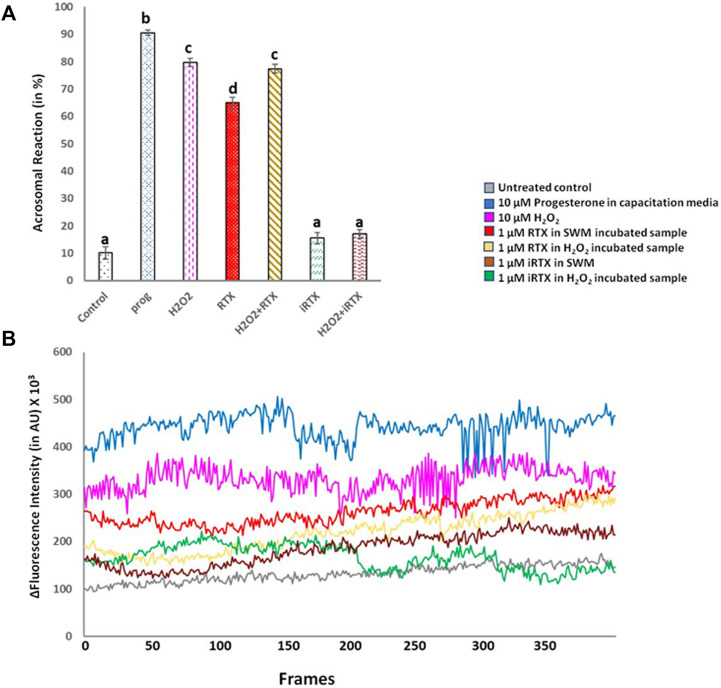
Effect of activation (by RTX) and inhibition (by iRTX) of TRPV1 on oxidatively stressed (H_2_O_2_-treated) spermatozoa, as compared to progesterone (prog) treated and untreated control (cnt) samples. **(A)** Bar graph comparing the percentage of spermatozoa with reacted acrosome for different treatment groups; data having superscripts of different letters are significantly different from each other. **(B)** Line graph showing the fluorescence intensities of [Ca^2+^]I concentrations (measured in arbitrary unit represented in %) at different frames of time-series imaging for different treatment groups, and the time difference between each frame is 5 s and frames 0–300 are shown here.

The acrosomal reaction was significantly augmented in H_2_O_2_-exposed spermatozoa; however, the level of increase was lesser than that of progesterone. RTX alone was able to induce an acrosomal reaction, but when administered in combination with H_2_O_2_, the level of induction was similar to H_2_O_2_ alone. iRTX did not affect acrosomal reaction at all, but brought back the acrosomal reaction induced by H_2_O_2_ to basal level (Figure 9A).

**FIGURE 10:**
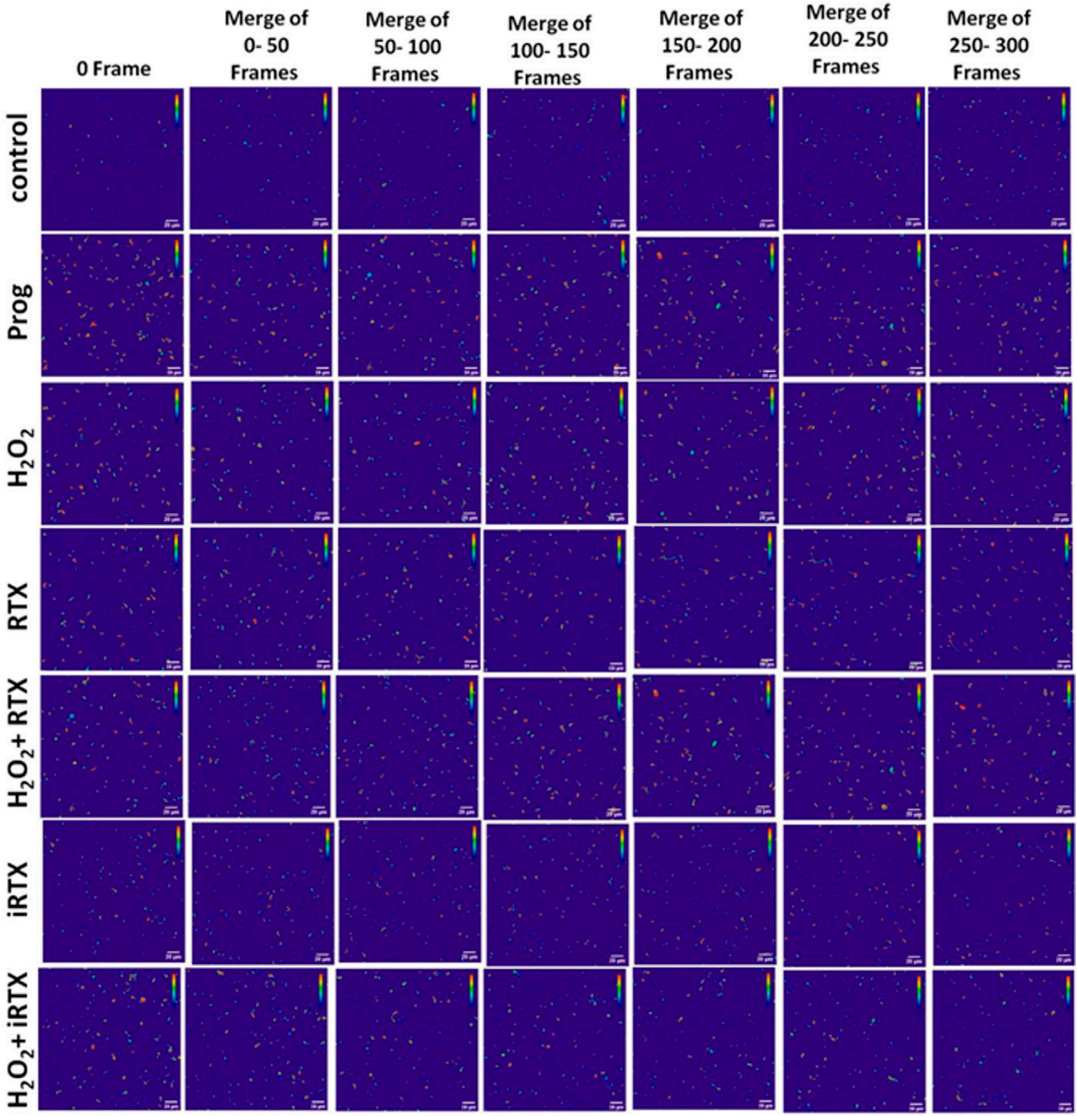
Time series images of Ca^2+^-intensity in live sperm cells. Cells were loaded with Fluo-4-AM and fluorescence intensity is represented in pseudo color (red and blue indicating highest and lowest intensity, respectively). Calcium imaging was performed at the speed of 2 frames per second.

Most acrosomal reaction-inducing agents (progesterone, RTX, and H_2_O_2_) caused an elevation of [Ca^2+^]i. Progesterone treatment resulted in the highest [Ca^2+^]i followed by H_2_O_2_ and then RTX. On the other hand, H_2_O_2_+RTX treatment showed a much lesser [Ca^2+^]i in comparison to control. iRTX alone increased the rate of [Ca^2+^]i for the initial 15 min, then brought the level back to below the control level by the end of the incubation period. When given in combination with H_2_O_2_, iRTX inhibited [Ca^2+^]i (Figure 9). Fluorescence intensity images derived from time-series imaging of view fields containing multiple cells for the different treated groups, loaded with Ca^2+^-sensing dye Fluo4-AM, were represented in Figure 10.

### TRPV1 shares an intricate relationship with curated candidate proteins involved in both natural and assisted fertilization success

In order to unravel the network affected by TRPV1 in conjunction with candidate proteins reported to regulate fertility success post natural conception or IVF, a secondary *in silico* data analysis was undertaken. Candidate proteins implicated to regulate successful fertility outcome post *in vivo/in vitro* fertilization were obtained by data mining and manual curation of published data. A total of seven reports were retrieved from the Pubmed/Medline^®^ database which documented differentially expressed proteins (DEPs) related to ART patients as explored by proteomic strategies. The studies were screened based on their study design and patient enrolment criteria. Studies that characterized DEPs related to pregnancy outcome post-IVF/ICSI exclusively identified by the LC/MS-MS approach were considered for secondary *in-silico* analysis. Studies were selected based on Preferred Reporting Items for Systematic Reviews and Meta-Analyses (PRISMA) criteria (Supplementary Figure S2). Out of the seven studies collected relevant to our study, four studies (Pixton et al., 2004; Frapsauce et al., 2014; Liu et al., 2018; Shen et al., 2019) were excluded for not meeting our criteria for a selection of DEPs. DEPs characterized by the following three studies, Zhu et al. (2013), Azpiazu et al. (2014), and Legare et al. (2014), were selected for the *in-silico* analysis with TRP channel proteins. Legare et al. (2014) enlisted 33 proteins to be differentially expressed in the sperm of men who did not achieve pregnancy by conventional IVF procedures as compared to fertile men. The remaining two studies (Zhu et al., 2013; Azpiazu et al., 2014) compared the sperm proteome profile by TMT-based LC-MS/MS, between men who did and did not achieve pregnancy with conventional IVF. The male partners of the aforementioned couples who achieved pregnancy by IVF can be thus presumed to be sub-fertile. The aforementioned three studies taken together enlisted the DEPs in sperm of men who did not achieve pregnancy by conventional IVF procedures as compared to fertile/subfertile men who achieved pregnancy by *in vivo/in vitro* fertilization. A total of 114 candidate proteins were thus characterized from sperm proteome, reported to influence successful fertility outcome post *in vivo/in vitro* fertilization (Supplementary Table S1).

**FIGURE 11:**
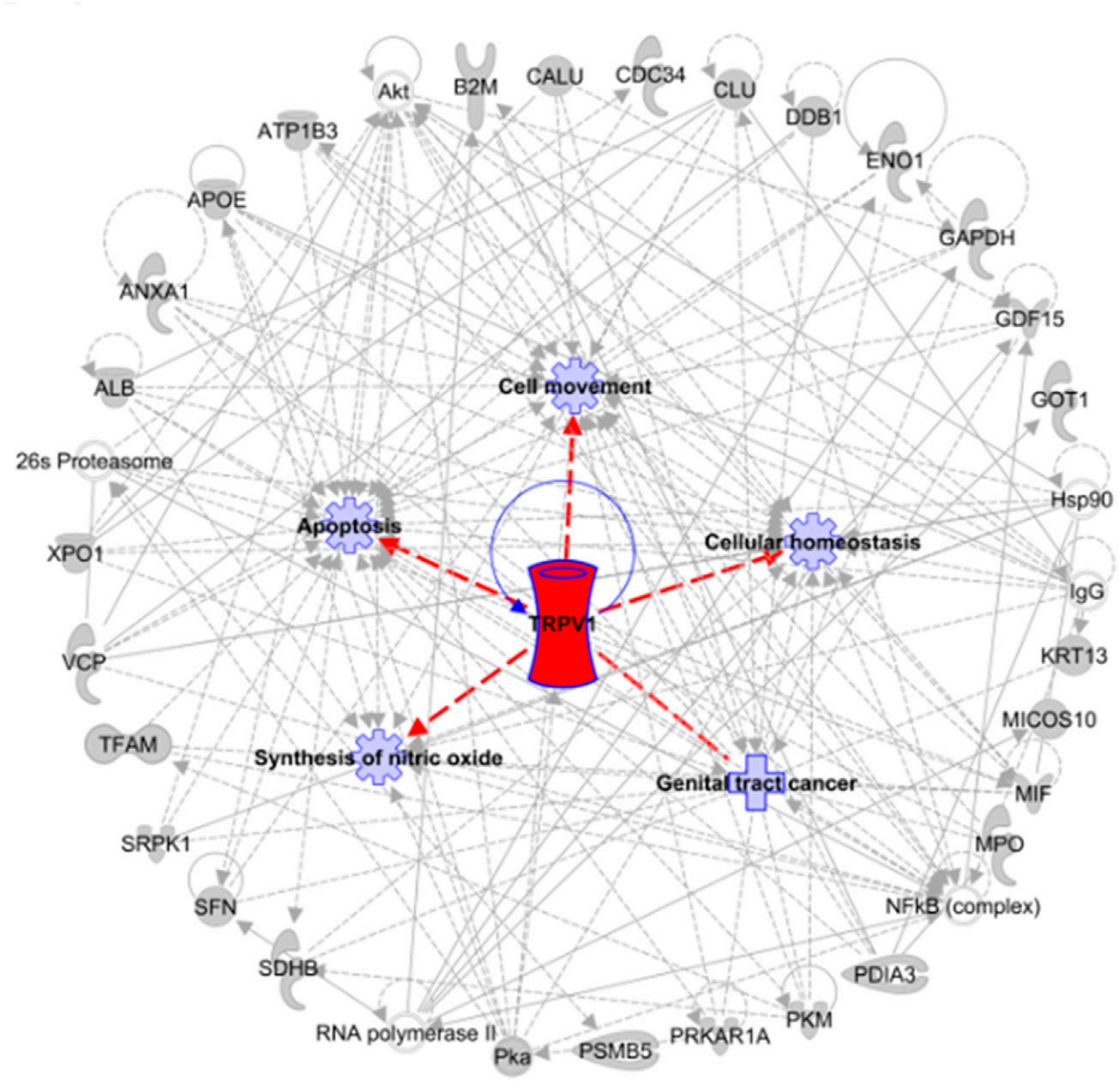
Ingenuity pathway analysis of the top disease and function networks affected by TRPV1 in conjunction with curated DEPs known to be associated with pregnancy outcome post ART a. Network 1: Cell death and survival, cellular compromise, hematological disease, embryonic development, and centered around TRPV1. Lines: interactions and arrowheads: directionality. The network was overlaid with the associated functions and diseases.

Genemania analysis from a curated database demonstrated the co-expression of TRPV1 with candidate proteins ankyrin repeat domain 28 (ANKRD28) and uncharacterized protein KIAA1683 (Ramaswamy et al., 2001; Noble et al., 2008). The biological interaction between the selected DEPs and TRPV1 relevant to fertility outcome post *in vivo*/*in vitro* fertilization was investigated using the IPA tool to outline the most enriched pathophysiological functions affecting reproductive success. With reference to the IPA curated disease ontology database, the most significant disease and functions associated with TRPV1 and DEPs included “Transmembrane potential of mitochondria” and “Cellular homeostasis” under the “Cell Morphology, Cellular Function and Maintenance” category. In concordance, Ingenuity Toxicity lists enlisted TRPV1, pyruvate kinase (PKM), and Beta-2-Microglobulin (B2M) to be associated with “mitochondrial dysfunction” (*p* = 4.03E-05). TRPV1 was found to be playing critical roles in developing neurological diseases in fetuses. The top scoring functional biological networks assigned to the selected DEPs and TRPV1 by IPA network analysis resulted in the identification of a network where 27 DEPs interacted with TRPV1, to regulate “Cell Death and Survival, Cellular Movement, Hematological Disease” (Figure 11, Supplementary Table S2).

## Discussion

The current study is targeted to observe the effect of differential expression of the heat-sensitive TRP channel TRPV1 in sperm on influencing fertility outcome via natural conception or conception with ART as well as in hypoxic and hyperthermic conditions (varicocele) with elevated ROS level. The selection of the respective TRP channel is based on its involvement with thermosensitivity and as oxidative stress signal transducers (Mori et al., 2016; Ogawa et al., 2016; Castillo, et al., 2018). Both conditions have been demonstrated to influence sperm quality and fecundity potential (Rao et al., 2016; Aitken and Drevet, 2020). Therefore, we further probed into the function of the channel in response to ROS (H_2_O_2_) exposure in the presence and absence of agonist and antagonist to validate its functional significance in human spermatozoa.

TRPV1 activity regulating sperm capacitation and acrosomal reaction has been well substantiated in different species (Maccarrone et al., 2005; Francavilla et al., 2009; Bernabò et al., 2010; Cobellis et al., 2010; Catanzaro et al., 2011; Gervasi et al., 2011; Lewis et al., 2012; Osycka-Salut et al., 2012; De Toni et al., 2016; Kumar et al., 2019). TRPV1 has been the most explored TRP channel in human sperm (Francavilla et al., 2009; Lewis et al., 2012; De Toni et al., 2016; Martínez-León et al., 2019) with functionality correlated to fertility, barring only one report (Mundt et al., 2018). The authors (Mundt et al., 2018) used TRPV1 agonist capsaicin which is 1,000–10,000 times less potent than RTX (Maggi et al., 1990) used in the present study and did not have an antagonist assay or peptide specificity assay. Therefore, they might have missed the localization of the channel in human sperm. The peptide control presented in this work regarding the used TRPV1 antibody is convincing and is further corroborated by the most potent TRPV1 agonist RTX and antagonist iRTX assay.

In the current study, the expression of the TRPV1 channel was probed in spermatozoa of male partners with successful pregnancy outcomes (fertile group) in comparison to those with unsuccessful pregnancy outcomes (infertile group) via natural/assisted reproduction in the phase I experiment for finding out the role of TRPV1 in imparting fertility potential to the spermatozoa. This is a pilot study in this field, which demonstrated that abnormal expression of the TRPV1 channel negatively affects the sperm quality as well as diminishes the fecundity potential of the sperm. Lewis et al. (2012) have demonstrated that functional loss of TRPV1 in spermatozoa of infertile men would eventually lead to fertilization failure due to abnormal capacitation and acrosomal reaction. Nevertheless, it is pertinent to mention here that knock-out models of TRPV1 animals are rarely sterile (Caterina et al., 2000; Colburn et al., 2007). Since TRPV1 channel activation is controlled by very diverse processes and, in many cases, exhibits complex polymodal properties, an analogy cannot be drawn between TRPV1 null mice and humans. Furthermore, natural selection always traded for the coexistence of redundant mechanisms, particularly for reproduction as a safety strategy wherein the failure of one of them does not paralyze the whole process (Bernabò et al., 2014). Hence, it may be the reason for the maintenance of fertility in TRPV1 KO mice where in absence of one ion channel (TRPV1) maintenance of fertility is taken care of by other redundant ion channels such as CatSper responsible for rheotaxisis under natural conditions (Miki and Clapham, 2013). It will not be out of context to mention here that in the knockout model system of another relatively close family member TRPV4, impairment of sperm’s migration ability, motility, and hyperactivation in response to thermotaxis was observed (Hamano et al., 2016) which may be responsible for decreased fertility. Similarly, binding of cannabinoids like anandamide and 2-arachibonoylglycerol to the TRPV1 and cannabinoids receptors (CB1 and CB2) in spermatozoa of fertile men was virtually absent for TRPV1 in spermatozoa of infertile men translating into a loss of function (capacitation, acrosomal reaction, and fertilization) in these patients (Lewis et al., 2012). However, the mRNA and protein levels remained stable in spermatozoa of the infertile patients. It is not inevitable to notice such changes in spermatozoa having limited capacity for transcription and translation. However, the functionality can be attributed to either translocation of the channel protein or its post-translational modification. The differential expression of TRPV1 in naturally fertile (NC+) and sub-fertile (IVF+ and ICSI+) may be attributed to its role in sperm motility and acrosomal reaction. In the case of IVF+ patients, where placing the spermatozoa in close proximity to the oocyte resulted in successful fertilization and embryo development, the role of TRPV1 may be attributed to its perfect acrosomal reaction and defective motility. In fact, in these patients, TRPV1 was predominantly located in the head implying its role in acrosomal reaction, but it is a significantly lower expression in the mid-piece and tail may be responsible for its decline motility making them incompetent under the natural condition to reach the egg up in the female reproductive tract (i.e., fallopian tube). Similarly, in the case of ICSI+ patients, its localization was predominantly in the mid-piece while very poor in the head region implying its lack of potency in acrosomal reaction; hence, natural fertilization was circumvented by intracytoplasmic injection into the oocyte. Therefore, the expression of TRPV1 in human spermatozoa may be a predictive factor for ART outcome. Within the female genital tract, sperm undergoes guided migration from the sperm deposition site to the fertilization site, which involves several tactic cues such as thermotaxis and chemotaxis. Remarkably, De Toni et al. (2016) observed that lower expression of TRPV1 impairs the ability of spermatozoa to migrate along a temperature gradient. The regulatory role of TRPV1 for the timely occurrence of acrosomal reaction was seen in other mammalian sperms (Bernabò et al., 2010; Kumar et al., 2019). Taking boar as the model system, it was explained that during capacitation, relocalization of TRPV1 occurs from post-acrosomal to the anterior region of the sperm head (Bernabò et al., 2008). TRPV1 was found to migrate to lipid microdomains of plasma membrane following bicarbonate-induced membrane reorganization, which might mediate the relocalization (Barbonetti et al., 2010). It was later demonstrated that exposure to TRPV1 agonist caused actin depolymerization in sperm, leading to loss of acrosomal integrity (Bernabò et al., 2010). Moreover, endocannabinoids preferentially anandamide have been shown to regulate the proper initiation and progression of capacitation and acrosomal reaction in sperm by the activating TRPV1 receptor in the female reproductive tract (Francavilla et al., 2009; Bernabò et al., 2010; Catanzaro et al., 2011; Gervasi et al., 2011; Kumar et al., 2019). Disruption of the above signaling cascades modulating motility, capacitation, and acrosomal reaction *in vivo*, possibly due to down-regulation of TRPV1 in these subfertile males would explain their need to undertake assisted techniques for successful fertilization *in vitro*.

Likewise, the differential expression of the TRPV1 channel has been reported to regulate the cellular response to oxidative stress and participate in stress-induced cellular damage (Bai and Lipski, 2010); nevertheless, a report on human spermatozoa is unavailable. The reduced expression of TRPV1 in spermatozoa of infertile patient samples with high ROS (ROS+) was reported for the first time. Lower expression of TRPV1 in these samples (ROS+) as compared to samples with low ROS (NC+), implied the possible involvement of TRPV1 in ROS signaling in sperm. This was further corroborated with the other major infertility condition varicocele. Spermatozoa are rich in polyunsaturated fatty acids which are more prone to systemic ROS attack resulting in a change in membrane fluidity (Agarwal et al., 2015; Aitken and Drevet, 2020) which might be responsible for the declined localization of the TRPV1 channel in spermatozoa membrane microdomain (Ramal-Sanchez et al., 2021) in these patients. In addition, estrogen-sensitive tissues like testis in TRPV1 KO mice are more susceptible to oxidative stress (Mizrak and Van Dissel-Emiliani, 2008). The lower levels of TRPV1 in spermatozoa of idiopathic males with high ROS could be attributed to irreversible oxidative modification of the channel and subsequent protein degradation as spermatozoa have limited capacity for transcription and translation. Moreover, Mizrak and Van Dissel-Emiliani (2008) have demonstrated the protective effect of TRPV1 in combating heat stress in male germ cells. It can be speculated that activation of TRPV1 might initiate downstream signaling pathways essential for germ cell survival under heat-stress conditions. Similarly, hypoxic activation of TRPV1 is also proposed to have physiological inference (Kim et al., 2012). Testicular and spermatozoal damage in unilateral varicocele patients has been shown to be due to hyperthermia and hypoxia-induced oxidative stress (Swain et al., 2020). Downregulation of TRPV1 in spermatozoa of these men would have aggravated the prevailing hyperthermic- and hypoxic-induced oxidative stress condition or *vice versa*.

In mature spermatozoa, TRPV1 was reported to be present in both head, mid-piece, and tail regions (De Toni et al., 2016; Kumar et al., 2019). Intracellular localization of TRPV1 was also proposed in spermatozoa (Maccarrone et al., 2005). We observed a similar expression pattern in the mature spermatozoa fraction. Immature spermatozoa having much lesser density (isolated from the 45% and 90% percoll gradient junction) than the mature ones (isolated as pellet penetrating 95% percoll) have a lower nucleus to cytoplasmic ratio. The retention of cytoplasm in these immature sperm cells due to aborted spermiogenesis may be responsible for the differential localization in subcellular cytoplasmic organelles increasing its intensity when measured by ICC. Similar results were also obtained with respect to abnormal spermatozoa with retention of residual cytoplasm. Previously, the co-expression of the channel in testicular tissues and their crosstalk has been proposed to regulate sperm development (Stein et al., 2004). Although TRPV1 mRNA and protein have been detected in spermatogonia, spermatocytes, and spermatids in mouse testis, spermatocytes were shown to have the highest expression of TRPV1 (Grimaldi et al., 2009). Therefore, its higher localization in immature cells is highly likely as it represents a partial developmental stage. Mizrak and van Dissel-Emiliani, have demonstrated TRPV1 knock-out models to be more susceptible to hyperthermia-induced testicular damage and consequent dysfunctional sperm development (Mizrak and Van Dissel-Emiliani, 2008). When the expression of TRPV1 in immature spermatozoa was studied by FACS, a completely reversed trend showing a declined expression with respect to mature sperms was observed. This may be due to the fact that during ICC preparation the spermatozoa are exposed to TritonX 100 making them permeable to the antibody for detection on intracellular proteins, while only the surface channels were detected in FACS.

TRPV1 is the only TRP channel reported so far to get activated within the redox potential range between −2000 mV and −3000 mV, making it one of the ideal redox sensors in cells (Mori et al., 2016). Oxidants and electrophiles generally have a redox potential threshold within this range. Redox sensing ability of TRPV1 has been corroborated in different cellular systems, where the channel is reported to get activated by ROS (Mukherjea et al., 2008; Yang et al., 2013; Johnstone et al., 2019). The co-localization of TRPV1 with NADPH oxidase 4 (NOX4) also speculated the intricate modulation of the channel function by intracellular H_2_O_2_ levels (Lin et al., 2015). Furthermore, the heat sensitivity of TRPV1 is ameliorated in dysfunctional redox conditions, indicating the modulation of the thermosensitive role of the channel in oxidative stress conditions (Vyklicky et al., 2002; Susankova et al., 2006). This is particularly important in the context of sperm migration in the female reproductive tract post-ejaculation. The involvement of multiple factors in synchronizing the timely occurrence of acrosomal reactions in mammalian sperm is undeniable. The cardinal role played by a minimal amount of ROS in this regard is pivotal and well documented. However, the identity and regulatory activity of key ion channels that act as downstream mediators complementing the physiological actions of ROS is not fully understood. The evidence obtained here is one such preliminary and novel study undertaken, demonstrating TRPV1 as a possible redox sensor and modulator in spermatozoa. The current findings suggest that H_2_O_2_ would initiate, regulate and synchronize key sperm functions by directly acting on TRPV1 as the downstream mediator. Exposure of spermatozoa to lower concentrations of H_2_O_2_ (1–10 µM) has been shown to regulate hyperactivated motility and acrosomal reaction (Oehninger et al., 1995). In other cellular models, TRPV1 channel activity due to the administration of agonist capsaicin was reported to be potentiated in presence of H_2_O_2_ (Demirdaş et al., 2017; Lam et al., 2018), while the effect of H_2_O_2_ could be reversed by treatment with TRPV1 antagonist capsazepine (Özdemir et al., 2016). However, we observed that the effect of H_2_O_2_ in inducing acrosomal reaction was attenuated in presence of TRPV1 antagonist 5′I-RTX implying the association of TRPV1 in mediating the H_2_O_2_ effect in spermatozoa. On the other hand, no significant potentiation of effect on the exposure of RTX to H_2_O_2_-treated sperm samples was noticed which could be due to the oxidation of thiol groups in RTX, compromising its binding to TRPV1. In fact, RTX binding to TRPV1 is reported to be thiol dependent and needs free SH groups for binding (Szallasi and Blumberg, 1993). In contrast, H_2_O_2_ mediates signaling by oxidizing the thiol groups on the redox-regulated proteins (Gutscher et al., 2009). In presence of H_2_O_2_, ligand binding would have been altered which has masked the potentiated effect of RTX in presence of H_2_O_2_ on sperm function, as observed here. Nevertheless, an increment in the percentage of sperm with acrosomal reaction was observed in both the treatment groups: H_2_O_2_ and H_2_O_2_ + RTX. The augmented Ca^2+^ influx due to channel activation by H_2_O_2_, both in the presence or absence of RTX, could be attributed to the major factor facilitating the aforementioned effect on the acrosomal reaction. An increase in intracellular calcium levels in response to TRPV1 channel activation have been previously demonstrated in human, boar, and bull sperm (Bernabò et al., 2010; De Toni et al., 2016; Gervasi et al., 2016). In concordance with these, a significant increment in Ca^2+^ was obtained for the two treatment groups, namely, H_2_O_2_ and H_2_O_2_ + RTX as compared to untreated groups, which was attenuated by incubating with iRTX. For the first time, we show that TRPV1 could be attributed to being one such calcium channel targeted by H_2_O_2_ to regulate Ca^2+^-dependent key sperm functions, especially acrosomal reaction. However, one interesting finding is the increase in [Ca^2+^]i in the spermatozoa of the iRTX-incubated (negative control) sample in absence of any stimulator till 15 min; thereafter, as expected, a sharp decline in [Ca^2+^]i was seen. It is generally believed and expected that activation of TRPV1 will increase intracellular Ca^2+^ and inhibition of TRPV1 will reduce the cytoplasmic Ca^2+^. TRPV1 is present in the endoplasmic reticulum (ER) and a number of reports suggest that this channel is involved in ER-mediated Ca^2+^-buffering (Haustrate et al., 2020). The sperm cell is an ER-free cellular system; hence, it could be possible that inhibition of TRPV1 may impair Ca^2+^-buffering leading to a rise in cellular Ca^2+^. Furthermore, iRTX binds to the TRPV1 at the same pocket as RTX, but with lesser affinity (Wahl et al., 2001). Therefore, the former may take a longer time to stabilize its interaction with the TRPV1 channel. Considering the sperm “lipidome” as a unique one and structurally much different than the somatic cells, it might be possible that TRPV1 in sperm has different pharmacological kinetics with respect to iRTX. Notably, previously, we reported increased motility of fish sperm in response to iRTX than in the control condition (Majhi et al., 2013).

Bibliographical studies have documented TRPV1 to be involved in various sperm functions ranging from acrosomal reactions, calcium trafficking, and fertilization; however, the contribution of the ion channel in determining the fecundity potential of sperm beyond fertilization awaits further exploration. The canonical pathways and networks related to sperm fertility, affected by these differentially expressed TRP channels, were further explored to understand their relevance to sperm physiology. As far as practicable, all the major female partners of the couples with confounding female factor abnormalities were eliminated in the current study, implying the restricted involvement of male factors in influencing the fertility outcome. Albeit the detailed correlation between TRP channel expression and prediction of pregnancy outcome remain indescribable, a pertinent explanation could be derived from the IPA results. Analysis of the crosstalk between TRPV1 with candidate proteins validated to influence fertility outcome showed that networks associated with cell death and survival, cellular compromise, hematological disease, and embryonic development were primarily affected by these TRP channels. The key biological disease and functions affected by TRPV1 in conjunction with the reported fertility proteins were apoptosis, cell movement, cellular homeostasis, genital tract cancer, and synthesis of nitric oxide (an important contributor to oxidative stress). The association of TRPV1 with tumorigenesis could be extrapolated to the regulation of cell division, especially during early embryonic development. Studies have demonstrated a great deal of similarity with regard to gene expression, epigenetic regulation, protein profiling, signaling pathways, and immunological aspects between embryogenesis and tumorigenesis (Ma et al., 2010; Manzo, 2019). Interestingly, TRPV1 activation has been demonstrated to stimulate notch and hedgehog/Wnt signaling as downstream pathways (Stock et al., 2014). Later is one of the key pathways characterized to be common between early embryogenesis and cancer (Kelleher et al., 2006).

The results of the present study surmise the role of TRPV1 in sperm function post ejaculation, particularly with respect to acrosome reaction and motility and at least partly toward Ca^+2^ homeostasis. The expression profile of TRPV1 may serve as an additional prognostic marker in ART outcome as well as a redox sensor in oxidative stress-mediated sperm dysfunction. However, further molecular studies involving miRNA-mediated TRPV1 regulation in spermatozoa may shed some light on its role. Because spermatozoa despite having limited transcriptional and translational activities are reported to be regulated by miRNA, particularly under asthenozoospermic, oligoasthenozoospermic, and/or tetrazoospermic patients compared to normozoospermic men, while several recent studies also report the regulation of TRPV1 by miRNAs (Ramal-Sanchez et al., 2021). This pilot study on the role of TRPV1 in sperm opens an arena for deciphering the scope of TRPV1 ligands and the interaction and binding sites, particularly in understanding sperm function (Fixemer et al., 2003; Puntambekar et al., 2005; Yang et al., 2018).

## Data Availability

The original contributions presented in the study are included in the article/Supplementary Material; further inquiries can be directed to the corresponding authors.

## Supplementary material

Supplementary Figure S1 Immunising peptide blocking assay to validate the effectiveness of the antibody.

Supplementary Figure S2 Preferred Reporting Items for Systematic Reviews and Meta-Analyses (PRISMA) flow diagram showing the search criteria for selection of studies enlisting the DEPs that determine the fertilization success post in vivo/ in vitro fertilization.
