# Supplementary material for: TRPV1 channel in spermatozoa is a molecular target for ROS-mediated sperm dysfunction and differentially expressed in both natural and ART pregnancy failure
Source: Front Cell Dev Biol. 2022 Sep 23;10:867057. doi: 10.3389/fcell.2022.867057 (PMC9538505; doi:10.3389/fcell.2022.867057)
Supplement: Supplementary file 1 [file Table1.docx]

**SUPPLEMENTARY TABLE**

**S1: List of candidate proteins selected from the reported studies for in silico analysis.**

| **Sl#** | **Protein** | **Uniprot No.** | **Gene name** | **Expression pattern** | **Reference** |
| --- | --- | --- | --- | --- | --- |
| 1 | AIG2-like domain 1/ Gamma-glutamylaminecyclotransferase | B0QY90 | GGACT | UE | Zhu et al. 2013 |
| 2 | Eukaryotic translation initiation factor 3 subunit l | P31947 | EIF3L | UE | Azpiazu et al. 2014 |
| 3 | Isoform 2 of 14-3-3 protein sigma | Q02383 | SFN | UE | Azpiazu et al. 2014 |
| 4 | Semenogelin-2 | Q9H0B3 | SEMG2 | UE | Azpiazu et al. 2014 |
| 5 | Uncharacterized protein KIAA1683/ IQ domain-containing protein N | B4DI70 | IQCN | UE | Legare et al. 2014 |
| 6 | cDNA FLJ53509, highly similar to Galectin-3-binding protein | Q6XZB0 |  | UE | Azpiazu et al. 2014 |
| 7 | Lipase member I | P02768 | LIPI | UE | Azpiazu et al. 2014 |
| 8 | Serum albumin | B3KRV7 | ALB | UE | Azpiazu et al. 2014 |
| 9 | Lipoprotein lipase | P54886 | LPL | UE | Azpiazu et al. 2014 |
| 10 | Isoform short of Delta-1-pyrroline-5-carboxylate synthase | O43592 | ALDH18A1 | UE | Azpiazu et al. 2014 |
| 11 | Exportin-T | Q6BCY4 | XPOT | UE | Azpiazu et al. 2014 |
| 12 | Isoform 2 of NADH-cytochrome b5 reductase 2 | Q99988 | CYB5R2 | UE | Azpiazu et al. 2014 |
| 13 | Growth/differentiation factor 15 | O75610 | GDF15 | UE | Azpiazu et al. 2014 |
| 14 | Left-right determination factor 1 | P0C7M6 | LFTY1 | UE | Azpiazu et al. 2014 |
| 15 | IQ domain-containing protein F3 | Q00059 | IQCF3 | UE | Azpiazu et al. 2014 |
| 16 | Transcription factor A, mitochondrial | B7Z1J9 | TFAM | UE | Legare et al. 2014 |
| 17 | Parkinson disease 7 domain-containing protein 1 | P15259 | PDDC1 | UE | Azpiazu et al. 2014 |
| 18 | Phosphoglycerate mutase 2 ( | Q8N427 | PGAM2 | UE | Legare et al. 2014 |
| 19 | Thioredoxin domain-containing protein 3 | Q96RL7 | TXND3 | UE | Azpiazu et al. 2014 |
| 20 | Isoform 4 of vacuolar protein sorting-associated protein 13A | Q5H943 | VPS13A | UE | Azpiazu et al. 2014 |
| 21 | Kita-kyushu lung cancer antigen 1 | P02649 | KKLC1 | UE | Azpiazu et al. 2014 |
| 22 | Apolipoprotein E | P35237 | APOE | UE | Azpiazu et al. 2014 |
| 23 | Serpin B6 | F5GX33 | SERPINB6 | UE | Azpiazu et al. 2014 |
| 24 | Calcium-binding mitochondrial carrier protein Aralar2 | O14980 | SLC25A13 | UE | Azpiazu et al. 2014 |
| 25 | Exportin-1 | O95336 | XPO1 | UE | Azpiazu et al. 2014 |
| 26 | 6-phosphogluconolactonase | P0CG04 | PGLS | UE | Azpiazu et al. 2014 |
| 27 | Ig lambda-1 chain C regions | Q92599 | IGLC1 | UE | Azpiazu et al. 2014 |
| 28 | Isoform 3 of Septin-8 | B4DY72 | SEPT8 | UE | Azpiazu et al. 2014 |
| 29 | Heat shock protein 105 kDa/ cDNA FLJ52360 | O15084 | Hsph1 | UE | Azpiazu et al. 2014 |
| 30 | Serine/threonine-protein phosphatase 6 regulatory ankyrin repeat subunit A | P51148 | ANKRD28 | UE | Azpiazu et al. 2014 |
| 31 | Ras-related protein Rab-5C | P28074 | RAB5C | UE | Azpiazu et al. 2014 |
| 32 | Proteasome subunit beta type-5 | Q96KX2 | PSMB5 | UE | Legare et al. 2014 |
| 33 | F-actin-capping protein subunit alpha-3 | B4DPM9 | CAPZA3 | UE | Azpiazu et al. 2014 |
| 34 | Serine hydroxymethyltransferase | P06733 | SHM1 | UE | Azpiazu et al. 2014 |
| 35 | Alpha-enolase | Q13561 | ENO1 | UE | Legare et al. 2014 |
| 36 | Dynactin subunit 2 | P09496 | DCTN2 | UE | Legare et al. 2014 |
| 37 | Clathrin light chain A | P28838 | CLCA | UE | Legare et al. 2014 |
| 38 | Cytosol aminopeptidase | P14314 | LAP3 | UE | Legare et al. 2014 |
| 39 | Glucosidase 2 subunit beta | Q9Y265 | PRKCSH | UE | Legare et al. 2014 |
| 40 | RuvB-like 1 | Q14257 | RUVBL1 | UE | Legare et al. 2014 |
| 41 | EF-hand calcium binding domain/ Reticulocalbin-2 | P21912 | RCN2 | UE | Zhu et al. 2013 |
| 42 | Succinate dehydrogenase [ubiquinone] iron-sulfur subunit, mitochondrial | P04406 | SDHB | UE | Legare et al. 2014 |
| 43 | Glyceraldehyde-3-phosphate dehydrogenase | P55072 | GAPDH | UE | Legare et al. 2014 |
| 44 | Transitional endoplasmic reticulum ATPase | Q9UK22 | VCP | UE | Legare et al. 2014 |
| 45 | F-box protein 2 | P49427 | FBXO2 | UE | Zhu et al. 2013 |
| 46 | Cell division cycle 34 homolog | Q712K3 | CDC34 | UE | Zhu et al. 2013 |
| 47 | ubiquitin-conjugating enzyme E2R2 | P06576 | UBE2R2 | UE | Zhu et al. 2013 |
| 48 | ATP synthase subunit beta, mitochondrial | P25705 | ATP5F1B | UE | Legare et al. 2014 |
| 49 | ATP synthase subunit alpha, mitochondrial | P48643 | ATP5F1A | UE | Legare et al. 2014 |
| 50 | T-complex protein 1 subunit epsilon | P50502 | CCT5 | UE | Legare et al. 2014 |
| 51 | Hsc70-interacting protein | O43852 | ST13 | UE | Legare et al. 2014 |
| 52 | Calumenin | P07205 | CALU | UE | Zhu et al. 2013 |
| 53 | Phosphoglycerate kinase 2 ( | P18754 | PGK2 | UE | Legare et al. 2014 |
| 54 | Regulator of chromosome condensation 1 | P54709 | RCC1 | UE | Zhu et al. 2013 |
| 55 | ATPase, Na+/K+ transporting, beta 3polypeptide | O00264 | ATP1B3 | UE | Zhu et al. 2013 |
| 56 | Membrane-associated Progesterone receptor membrane component 1 | O15173 | PGRMC1 | UE | Zhu et al. 2013 |
| 57 | Membrane-associated Progesterone receptor membrane component 2 | Q8TCS8 | PGRMC2 | UE | Zhu et al. 2013 |
| 58 | Polyribonucleotide nucleotidyltransferase 1 | O00154 | PNPT1 | UE | Zhu et al. 2013 |
| 59 | Acyl-CoA thioesterase 7/ Brain acyl-CoA hydrolase | P26436 | ACOT7 | UE | Zhu et al. 2013 |
| 60 | Acrosomal vesicle protein 1 | Q8N0W7 | ACRV1 | UE | Zhu et al. 2013 |
| 61 | Fragile X mental retardation 1 neighbor | P16562 | FMR1NB | UE | Zhu et al. 2013 |
| 62 | Cysteine-rich secretory protein 2 | P54108 | CRISP2 | UE | Zhu et al. 2013 |
| 63 | Cysteine-rich secretory protein 3 | P49221 | CRISP3 | UE | Zhu et al. 2013 |
| 64 | Protein-glutamine gamma-glutamyltransferase 4 | P14618 | TGM4 | OE | Legare et al. 2014 |
| 65 | Pyruvate kinase PKM | P35579 | PKM | OE | Legare et al. 2014 |
| 66 | Myosin-9 | P06744 | MYH9 | OE | Legare et al. 2014 |
| 67 | Glucose-6-phosphate isomerase | P30101 | GPI | OE | Legare et al. 2014 |
| 68 | Protein disulfide-isomerase A3 | P08910 | PDIA3 | OE | Legare et al. 2014 |
| 69 | Abhydrolase domain containing 2 | P08237 | ABHD2 | OE | Zhu et al. 2013 |
| 70 | Phosphofructokinase, muscle | O14556 | PFKM | OE | Zhu et al. 2013 |
| 71 | Glyceraldehyde-3-phosphate dehydrogenase, testis-specific | P62805 | GAPDHS | OE | Legare et al. 2014 |
| 72 | Histone H4 | P10909 | H4C | OE | Azpiazu et al. 2014, Legare et al. 2014 |
| 73 | Clusterin | O76024 | CLU | OE | Legare et al. 2014 |
| 74 | Wolframin | F8WAS3 | WFS1 | OE | Azpiazu et al. 2014 |
| 75 | NADH dehydrogenase [ubiquinone] 1 alpha subcomplex subunit 5 | O14949 | NDUFA5 | OE | Azpiazu et al. 2014 |
| 76 | Cytochrome b-c1 complex subunit 8 | Q5T5M1 | UQCRQ | OE | Azpiazu et al. 2014 |
| 77 | Aquaporin 7 | P61769 | AQP7 | OE | Azpiazu et al. 2014 |
| 78 | Beta-2-microglobulin | O14841 | B2M | OE | Zhu et al. 2013 |
| 79 | 5-oxoprolinase | Q96QV6 | OPLAH | OE | Azpiazu et al. 2014 |
| 80 | Histone H2A type 1-A | Q16531 | HIST1H2AA | OE | Azpiazu et al. 2014 |
| 81 | DNA damage-binding protein 1 | Q93077 | DDB1 | OE | Legare et al. 2014 |
| 82 | Histone H2A type 1-C | P14174 | HIST1H2AC | OE | Azpiazu et al. 2014 |
| 83 | Macrophage migration inhibitory factor | P49721 | MIF | OE | Azpiazu et al. 2014 |
| 84 | Proteasome subunit beta type-2 | O43464 | PSMB2 | OE | Azpiazu et al. 2014 |
| 85 | Isoform 3 of serine protease HTRA2, mitochondrial | Q16777 | HTRA2 | OE | Azpiazu et al. 2014 |
| 86 | Histone H2A type 2-C | Q96IX5 | HIST2H2AC | OE | Azpiazu et al. 2014 |
| 87 | Up-regulated during skeletal muscle growth protein 5 | P53041 | USMG5 | OE | Azpiazu et al. 2014 |
| 88 | Protein phosphatase 5 | E5RJX2 | PPP5C | OE | Zhu et al. 2013 |
| 89 | 40S ribosomal protein S20 | P13646 | RPS20 | OE | Azpiazu et al. 2014 |
| 90 | Isoform 3 of Keratin, type I cytoskeletal 13 | Q5TGZ0 | KRT13 | OE | Azpiazu et al. 2014 |
| 91 | Mitochondrial inner membrane organizing system protein 1 | E7EPB3 | MICOS10 | OE | Azpiazu et al. 2014 |
| 92 | 60S ribosomal protein L14 | Q96SB4 | RPL14 | OE | Azpiazu et al. 2014 |
