# Supplementary figures and images for: TRPV1 channel in spermatozoa is a molecular target for ROS-mediated sperm dysfunction and differentially expressed in both natural and ART pregnancy failure
Source: Front Cell Dev Biol. 2022 Sep 23;10:867057. doi: 10.3389/fcell.2022.867057 (PMC9538505; doi:10.3389/fcell.2022.867057)

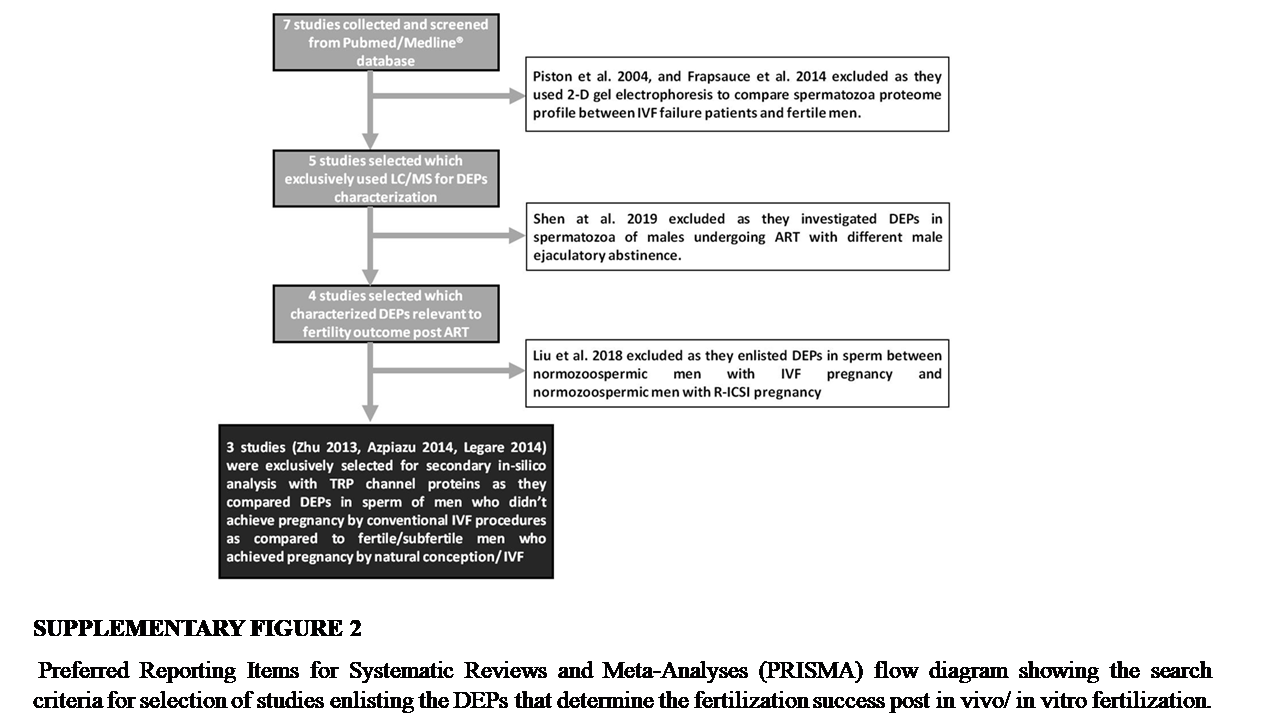

Supplement: Supplementary file 2 [file Image2.tif]

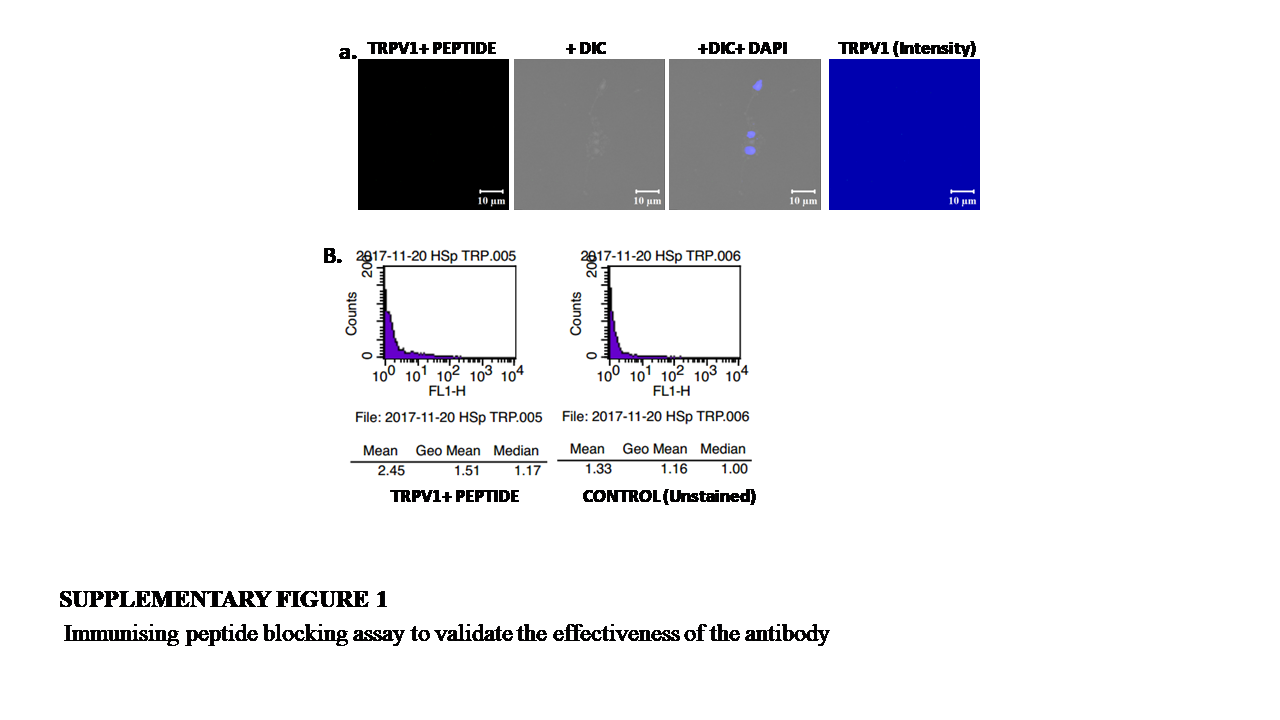

Supplement: Supplementary file 3 [file Image1.tif]
