# Supplementary material for: TRPV1 channel in spermatozoa is a molecular target for ROS-mediated sperm dysfunction and differentially expressed in both natural and ART pregnancy failure
Source: Front Cell Dev Biol. 2022 Sep 23;10:867057. doi: 10.3389/fcell.2022.867057 (PMC9538505; doi:10.3389/fcell.2022.867057)
Supplement: Supplementary file 4 [file Table2.docx]

**SUPPLEMENTARY TABLE**

S2: Involvement of TRPV1 in the top disease and function networks associated with sperm proteome dataset predicted to influence pregnancy outcome post IVF as compared to fertile/ subfertile males. All of the molecules that compose each network are listed.The score is based on a p-value calculation, which calculates the likelihood that the Network Eligible Molecules that are part of a random chance alone. Focus Molecules column simply indicates the number of Network Eligible Molecules per network.

| **ID** | **Molecules in Network** | **Score** | **Focus Molecules** | **Top Diseases and Functions associated** |
| --- | --- | --- | --- | --- |
| **1** | **TRPV1,** 26s Proteasome, Akt,ALB,ANXA1,APOE,ATP1B3,B2M,CALU,CDC34,CLU,DDB1,ENO1,GAPDH,GDF15,GOT1,Hsp90,IgG,KRT13,MICOS10,MIF,MPO,NFkB (complex),PDIA3,Pka,PKM,PRKAR1A,PSMB5,RNA polymerase II,SDHB,SFN,SRPK1,TFAM, ,VCP,XPO1 | 56 | 28 | Cell Death and Survival, Cellular compromise, Hematological Disease, Embryonic development |
