# Supplementary material for: TRPV1 channel in spermatozoa is a molecular target for ROS-mediated sperm dysfunction and differentially expressed in both natural and ART pregnancy failure
Source: Front Cell Dev Biol. 2022 Sep 23;10:867057. doi: 10.3389/fcell.2022.867057 (PMC9538505; doi:10.3389/fcell.2022.867057)
Supplement: Supplementary file 5 [file DataSheet1.docx]

**Supplementary 1 - Details of the methodology**

**S1.1 *Measurement of reactive oxygen species (ROS) in sperm by luminol-dependant chemiluminescence assay***

Sperm pellet extracted from semen, was resuspended in the sperm washing media and used for analysis. ROS formation was measured by chemiluminescence assay using luminol (5-amino-2, 3-dihydro-1, 4-phthalazinedione) as the probe. Luminol is an extremely sensitive dye that detects both extracellular and intracellular ROS at neutral pH. Luminol generates light signal after combining with oxidative end products, which is measured with a luminometer.

The liquefied semen was centrifuged at 300 g for 7 minutes to separate the sperm from seminal plasma. The sperm pellet was washed with phosphate-buffered saline (PBS) and resuspended in the sperm washing media. For measuring ROS, luminol was added in working solution to 400 μl sperm suspension. The working solution of luminol (Sigma-Aldrich, UK) is 5 mM prepared in dimethylsulphoxide (DMSO), and the concentration of H202 (VWR UK) used for positive control is 30% in DW. The assay mixture consisted of only PBS (400 μl) for the blank; PBS (400 μl) + 5 mM luminol (10 μl) for the negative controls; PBS (395 μl) + 30 % H202 (5 μl ) + of 5 mM luminol (10 μl ) for the positive controls; sperm suspension (400 μl) + 5 mM luminol (10 μl) for test sample. All samples were vortexed gently immediately and levels of ROS were assessed by measuring the luminol-dependant chemiluminescence with the Berthold luminometer (Model: AutoLumat plus LB 953, Oakridge, TN). ROS of the test sample was calculated by subtracting the negative control average from its average. The sample ROS is then corrected by dividing it with “sperm concentration/mL. Results were expressed as relative light units RLU/s/million sperm/ ml.

Sample ROS = average “RLU mean” for sample - average “RLU mean” for negative control.

Corrected sample ROS = Calculated sample ROS/sperm concentration = XX.X (RLU/s/million sperm/ml).

**S1.2 *Intensity quantification of confocal images of single sperm cell using FIJI software***

The confocal image was opened in Fiji software in Composite colour mode. Images are then convertedto a stack with the menu command: “Image/Stacks/Z project” with projection type as maximum intensity. The channels were split using the menu command: Image/Color/Split Channel. The image with only DIC channel was selected, the sperm cell of interest was drawn using free hand selection tool and added as region of interest (ROI) using the menu command: Analyse/Tool/ROI manager/Add. The image with targeted channelwas selected (in this case green) and the uneven backgroundwas removed using Rolling ball plugin with menu command: Process/Subtract background/ sliding paraboloid(default radius 50 pixels). The background corrected image was selected and for thresholding correction using the menu command: Image/Lookup Tables/HiLo, followed by Image/Adjust/Brightness/Contrast, the “Minimum” slider of brightness/ contrast was adjusted until the entire background is blue and applied. Thresholding is done to distinguish the background from the foreground. The ROI from the ROI manager was then added to the background corrected image and the intensity was measured using menu command: Analyse/measure. Prior to measurements, the area, integrated density and mean gray value were set to be measured using menu command: Analyse/set measurements.For normalization, a region with no fluorescence next to sperm cell with same area (same ROI) is taken as background.The measurements were then copied to Microsoft Excel datasheet and the corrected total cell fluorescence (CTCF) was calculated using the following formula: CTCF = Integrated Density - (Area of selected cell X Mean gray value of background readings).

**S1.3 *Intensity quantification of time-series images showing Ca^2+^-influx by sperm cells using FIJI software***

The time series confocal image was opened in Fiji softwareand the channels were split using the menu command: Image/Color/Split Channel. DIC image was closed and the image with targeted channel was selected (in this case green). The image is converted to 8-bit grayscale using command Edit/Options/Conversions/scale. The uneven background was removed using Rolling ball plugin with menu command: Process/Subtract background/ sliding paraboloid(default radius 50 pixels). The background corrected image was selected and automaticthresholding correctionwas carried out using menu command: Image/Adjust/Threshold and threshold was adjusted in B/W mode to remove background noise. For automatically separating or cutting apart particles that touch each other, watershed segmentation is undertakenusing menu command: Process/Binary/Watershed. The entire image was selected by rectangle selection tool and added as ROI using the menu command: Analyse/Tool/ROI manager/Add. The intensity of all the frames were calculated with menu command: Tool/ROI manager/More/multimeasure.
